# Feed Additives Differentially Impact the Epimural Microbiota and Host Epithelial Gene Expression of the Bovine Rumen Fed Diets Rich in Concentrates

**DOI:** 10.3389/fmicb.2020.00119

**Published:** 2020-02-19

**Authors:** Renee Maxine Petri, Viktoria Neubauer, Elke Humer, Iris Kröger, Nicole Reisinger, Qendrim Zebeli

**Affiliations:** ^1^Institute of Animal Nutrition and Functional Plant Compounds, University of Veterinary Medicine, Vienna, Austria; ^2^Institute for Food Safety, Food Technology and Veterinary Public Health – Unit for Food Microbiology, Department for Farm Animals and Veterinary Public Health, University of Veterinary Medicine, Vienna, Austria; ^3^FFoQSI GmbH – Austrian Competence Centre for Feed and Food Quality, Safety and Innovation, Tulln, Austria; ^4^BIOMIN Research Center, BIOMIN Holding GmbH, Tulln, Austria

**Keywords:** feed additives, phytogenics, autolyzed yeast, rumen epithelial microbiome, gene expression

## Abstract

The success of nutritional strategies for the prevention of subacute ruminal acidosis (SARA) and the related microbial dysbiosis still remains unpredictable due to the complexity of the rumen ecosystem. The rumen epimural community, due to proximity, has the greatest opportunity to influence host gene expression. The aim of this study was to determine the effect of two separate feed additives on the rumen epimural community and host epithelial gene expression. Eight rumen cannulated Holstein cows were randomly assigned to one of three feeding groups: autolyzed yeast (AY), phytogenics (PHY) and control (CON) using a 3 × 3 Latin square design. Cows were fed an intermittent SARA model that started with 100% forage diet (Baseline) followed by two 65% concentrate-diet induced SARA challenges (SARAI, SARAII), separated by 1 week of forage only feeding. Rumen papillae samples were collected via the cannula during the Baseline, SARAI and SARAII periods. Microbial DNA was extracted and sequenced targeting the 16S rRNA gene and host RNA was analyzed using RT-qPCR. Analysis of the taxonomic composition at the genera level showed a tendency to increase in the relative abundances of *Pseudobutyrivibrio* (*P* = 0.06), *Selenomonas* (*P* = 0.07) and significantly increase in SHD-231 (*P* = 0.01) in PHY treated animals, whereas *Succiniclasticum* tended to decrease in both PHY and AY treated animals compared to the control. Linear discriminant analysis effect size testing was performed and based on treatment × feeding phase interaction, a number of biomarker genera were identified including the previously identified *Succiniclasticum*. Supplementation with AY correlated positively with *CD14* and *DRA* expression and negatively to *CLDN1*, *MyD88*, and *MCT4* expression. Supplementation with PHY showed a negative correlation to *CLDN4* gene expression. *Anaerovibrio* showed the highest positive Pearson correlations to biogenic amines tested in the rumen fluid including putrescine (*r* = 0.67), cadaverine (*r* = 0.84), and tyramine (*r* = 0.83). These results show that supplementing feed additives to high grain diets can have a positive influence on the stability of the epimural populations, and that changes in the epimural community are correlated with changes in host epithelial gene expression.

## Introduction

The rumen is a complex microbial ecosystem in the foregut of ruminants, which is evolutionarily adapted to a fiber-rich diet, allowing them to utilize complex carbohydrate sources not suitable for human-nutrition. Yet, the modern production practice of feeding large amounts of concentrate for increased physiological energy requirements can disrupt this ecosystem resulting in microbial dysbiosis. This dysbiosis is commonly described as subacute ruminal acidosis (SARA), defined as extended periods of time (>5 h/day) where ruminal pH is below physiological range (5.8; Zebeli et al., 2008). This results in changes in ruminal microbial populations including decreased diversity (Petri et al., 2013), altered fermentation patterns (Kleen et al., 2003), as well as increased free endotoxin release (Plaizier et al., 2012), altered epithelial uptake and metabolism (Steele et al., 2011, 2012), and affect animal health (Zebeli and Metzler-Zebeli, 2012). Previously, ionophores (monensin, lasalocid) and other rumen stabilizing antibiotics were used to prevent this ecological dysbiosis (Duffield and Bagg, 2000). However, as antibiotics are being phased out of livestock production, alternative strategies must be developed and tested to evaluate possible methods of modulating rumen microbial fermentation during high-concentrate feeding. The use of bioactive phytochemicals as natural feed additives has recently gained interest as an antibiotic alternative for modifying rumen fermentation favorably, such as by minimizing rumen methanogenesis and improving rumen fermentation (Flachowsky and Lebzien, 2012). Similarly, prebiotics such as autolyzed yeast have also been used to increase fiber and starch digestion (Harrison et al., 1988; Mao et al., 2013), to prevent rumen acidosis (Nocek et al., 2011; Ganner and Schatzmayr, 2012), and to accelerate rumen microbial biofilm development (Chaucheyras-Durand and Fonty, 2002). However, the effectiveness of these nutritional interventions is highly variable depending upon the interactions among the chemical structure of the supplement, the diet, and the adaptability of the rumen microbiota.

We have previously reported the effects of phytogenic and yeast products on the rumen fermentation (Kröger et al., 2017), as well as changes in digesta and liquid associated populations under intermittent high-grain feeding programs (Neubauer et al., 2018). We noted that the feeding of these feed additives reduced rumen and fecal associated toxins including biogenic amines and lipopolysaccharide (Humer et al., 2018). However, previous research has also shown that despite the changes of the digesta associated microbiota, the epithelial-associated microbe population can remain stable (Petri et al., 2013), and have also been shown to be correlated to rumen papillae gene expression associated with short-chain fatty acids (SCFA) absorption and pH regulation (Petri et al., 2019). However, the impact of feed additives on epithelial microbiota, host physiology, and the associations between the host and microbes to rumen endotoxin levels under SARA conditions remains largely unknown. Thus, understanding the interactions between the gut microbiota, diet, and host response are key to develop new strategies for reducing dysbiosis, and rumen epithelial function, thereby improving rumen health and efficiency.

The aim of this study was to determine the effect of supplementation of PHY or AY feed additives under repeated bouts of diet induced SARA on rumen epithelial (i.e., epimural) microbial populations, and host papillae gene expression, as well as their interaction with regards to barrier function, and pro-inflammatory signaling. Our hypothesis was that PHY and AY will reduce dysbiosis in the rumen by supporting the epithelial associated bacterial community, increasing barrier function gene expression, and reducing TLR4-signaling, especially during the first high grain feeding, since the duration of low pH was longer during this phase (Kröger et al., 2017). We also hypothesized that the supplementation of feed additives would reduce the amount of rumen toxins positively correlated with epithelial microbiota growth and host barrier function gene expression.

## Materials and Methods

All procedures involving animal handling and treatment were approved by the institutional ethics committee of the University of Veterinary Medicine Vienna and the national authority according to section 26 of the Law for Animal Experiments (GZ: BMWFW-68.205/0023-WF/V/3b/2015).

### Experimental Design and Sample Collection

Detailed information about animals, feeding, and experimental setup is given in Kröger et al. (2017). In brief, eight non-lactating rumen-fistulated Holstein-Friesian cows (863 ± 65 kg BW ± SD) were used in an incomplete 3 × 4 Latin square design balanced for carry-over effects. Cows were randomly assigned to one of the three feeding groups: CON (Control, no feed additive), PHY (Digestarom^®^ Dairy BIOMIN Holding GmbH, Inzersdorf-Getzersdorf, Austria; 3 g per cow per day) and AY (Levabon^®^ Rumen E, BIOMIN Holding GmbH, Inzersdorf-Getzersdorf, Austria; 15 g per cow per day). The PHY consisted of a blend of spices, herbs, and essential oils, whereas AY contained autolyzed spray-dried yeast (*Saccharomyces cerevisiae*). Each of the four periods included 1 week forage feeding (100% hay diet, RD; Baseline), 6 days of gradual concentrate adaptation, 1 week high-concentrate challenge (65% concentrates, in DM basis, SARAI), then 1 week of roughage only (recovery phase), followed by a second 65% concentrate challenge lasting 2 weeks (SARAII) based on previously established SARA models (Qumar et al., 2017). Feed additives were provided in the concentrate mix except during the recovery phase when no concentrate was fed, then daily doses of PHY and AY were provided via the rumen cannula. No supplements were fed in the Baseline period. Each experimental period was followed by a 3 week long washout phase with roughage only to avoid carry-over effects of high-grain feeding and feed additives.

The forage diet consisted of a 50:50 ratio (DM basis) of grass silage and hay, and the concentrate mixture contained barley grain (33%), wheat (30%), rapeseed meal (16%), corn (15%), beet pulp (3.2%), a mineral-vitamin premix (1%; containing 13.5% Ca; 9% Mg; 5% P; 1.5% Na; 2,100,000 IU vitamin A/kg; 300,000 IU vitamin D/kg; 7,500 mg vitamin E/kg), beet molasses (1%), calcium carbonate (0.5%), and NaCl (0.3%) on DM basis (Kröger et al., 2017). The TMR diet fed through the SARA challenges consisted of 29.2% NDF and 32.3% starch, on a DM basis. Diets were fed *ad libitum* with a 10% orts refusal and offered via individual feeding troughs equipped with computer-controlled electronic scales. Access was regulated electronically with transponder access gates (Insentec B.V., Marknesse, Netherlands). Samples of rumen fluid were collected on d 6 (Baseline), 19 (SARAI), and 39 (SARAII) 8 h after morning feeding and analyzed for biogenic amines as described in Humer et al. (2018). The method to collect particle-associated rumen liquid for determination of biogenic amines and LPS has been previously described (Humer et al., 2018). Briefly, approximately 500 g of rumen digesta was taken from the middle of the rumen mat and squeezed through 4 layers of cheesecloth to obtain 250 mL of rumen liquid, which was stored at −20°C before analysis.

### Rumen Papilla Biopsies

Rumen papillae biopsies for sequencing and gene expression approaches were taken on day 7 (Baseline), 20 (SARAI), and 40 (SARAII) 2 h (1000 h) after the morning feeding. The technique was previously described by Wetzels et al. (2017). Briefly, the withdrawn rumen content was kept in a bucket in a 39°C water bath in order to avoid cooling and put back in after the sampling procedure. The rumen was partly evacuated so that the wall of ventral rumen sac could be lifted through the rumen cannula. Biospies were taken from 40 to 50 cm below the bottom edge of the rumen cannula located in the left fossa paralumbalis (Wetzels et al., 2017). The epithelium was thoroughly rinsed with sterile phosphate-buffered saline (PBS) solution until no digesta particles were left and epithelial tissue was sampled using disinfected scissors and tweezers. Each of the biopsies was quickly rinsed with sterile PBS to remove contaminants, shock frozen in liquid nitrogen, transferred into cryotubes (Sarstedt AG, Wiener Neudorf, Austria), and stored at −80°C for DNA and RNA extraction.

### DNA-Extraction and Sequencing

Rumen papillae biopsies were partially thawed on ice and genomic DNA was extracted from 0.25 g rumen papillae, using a sample preparation protocol and the PowerSoil^®^ DNA Isolation Kit (MO BIO Laboratories, Inc., Carlsbad, CA, United States) as previously described by Neubauer et al. (2019). After DNA extraction, samples were stored at −20°C. Total DNA quantity after isolation was measured for all samples using Qubit Fluorometer 2.0 (Qubit dsDNA HS Assay Kit, Thermo Fisher Scientific, Vienna, Austria) according to manufacturer’s instructions. Amplicon sequencing was performed using Illumina MiSeq paired-ends sequencing technology (Microsynth AG, Balgach, Switzerland). The hypervariable region V3–V5 of bacterial 16S rRNA genes was amplified by bridge amplification using the primer set 357F (5′-CCTACGGGAGGCAGCAG-3′), and 926R (5′-CCGTCAATTCMTTTRAGT-3′; Peterson et al., 2009), to generate an approximate amplicon size of 570 bp. Multiplexed libraries were constructed by ligating sequencing adapters and indexes onto purified PCR products using the Nextera XT Sample Preparation Kit (Illumina, Balgach, Switzerland). Primers were trimmed and corresponding overlapping paired-end reads were stitched by Microsynth (Microsynth AG, Balgach, Switzerland; Bokulich et al., 2013). Sequence data were analyzed with the open source bioinformatics pipeline QIIME (version 1.9.1.)^1^, based on the recommended workflow of QIIME tutorials^2^ (last access January, 2017; Caporaso et al., 2010) and Navas-Molina et al. (2013). Briefly, sequences of low quality were trimmed with a quality score of 20. The chimeric sequences were identified using the gold.fa reference database (Edgar, 2010), and subsequently filtered.

A total of 817,138 sequences passed the quality control and chimera check. Sequences were then clustered into operational taxonomic units (OTUs) with a 97% 16S rRNA gene similarity cut-off by performing open reference OTU picking. The database of SILVA^3^ (version 123; Quast et al., 2012) was used for taxonomic classification. A total of 1150 OTUs were found, with an average of 9,948 sequences per sample. The percent relative abundances of all OTUs were calculated and OTUs were ranked according to their abundance. Measures of alpha diversity were also determined using QIIME, specifically non-parametric species richness estimator Chao1, Shannon, and Simpson Indices, as well as number of observed OTUs per sample. The rarefaction depth was equalized for all samples to 344 sequences per sample based on the minimum sequence number achieved. Goods coverage rarefaction curves for the sampling depth during the feeding phases, as well as for the feed additive groups is shown in Supplementary Figure 1. Sequencing results were analyzed at phyla and genus level as a percent of relative abundance. For interpreting changes of the highly dominant taxa, the most abundant 100 OTU with their taxonomic assignment of ≥ 97% sequence similarity to SILVA or NCBI were chosen. The linear discriminant analysis effect size (LEfSe) (Segata et al., 2011) analysis was used to identify specific OTUs that differed between treatments (CON, PHY, and AY) and feeding phase (Baseline, SARAI, and SARAII). LEfSe uses a non-parametric factorial Kruskal–Wallis sum-rank test followed by a linear discriminate analysis to identify both statistically significant and biological relevant features. The OTU relative abundances were used as an input for LEfSe^4^ (last accessed October, 2018) following the methodology of Segata et al. (2011). Bray Curtis distance matrixes, non-metric multidimensional scaling analysis, and canonical correspondence analysis (CCA) were done using the package Vegan in R (Oksanen et al., 2018).

The sequencing data were deposited into the European Nucleotide Archive (ENA) and can be accessed via accession numbers PRJEB33839 and PRJEB29866 for CON samples.

### RNA Isolation and Reverse Transcription qPCR

For RNA isolation, the method previously described by Petri et al. (2019) was used. Briefly, 20 mg of papillae from each animal were combined with Lysis buffer (RNease Mini QIAcube kit, Qiagen, Hilden, Germany) and autoclaved ceramic beads (0.6 g; 1.4 mm; VWR), and homogenized 6.5 ms-1 for 30 s in a FastPrep-24 instrument (MP Biomedicals, Santa Ana, CA, United States). Then samples were placed in the QIAcube robotic workstation (Qiagen, Hilden, Germany) to complete RNA extraction according to the manufacturer. Genomic DNA was then digested (Turbo DNA kit, Life Technologies Limited, Vienna, Austria) and RNA concentration was measured (Qubit HS RNA Assay kit, Qubit 2.0 Fluorometer, Life Technologies), and quality tested (Agilent RNA 6000 Nano Assay Kit, Agilent Bioanalyzer 2100, Agilent Technologies, Waghäusel-Wiesental, Germany). The mean RNA integrity (RIN) value for all samples was 7.9 ± 0.67, with two samples having RIN values below 7.0 (5.6 and 6.2), and the other samples ranging between 7.0 and 9.2. Complementary DNA (cDNA) was synthesized (High Capacity cDNA RT kit, Life Technologies Limited, Vienna, Austria) from 2 μg RNA in duplicate using a 2-step PCR program (Mastercycler nexus, Eppendorf, Hamburg, Germany) according to the previously published method (Petri et al., 2019), using an incubation at 25°C for 10 min, reverse transcription at 37°C for 120 min, and a final heating step at 85° for 5 min. Reverse transcription controls (1 μl × 1 μl) were included as a control for residual DNA contamination. Each sample was analyzed in duplicate, reverse transcription controls and negative controls were included in duplicates as well.

For gene expression analysis, qPCR reactions were conducted using the following thermal program: at 95°C for 5 min, and 40 cycles of 95°C for 10s melting, 60°C for 30 s annealing, and 72°C for 30 s final elongation (Mx3000P thermocycler, Agilent Technologies). Melting curve analysis was completed to determine primer specificity (Petri et al., 2019). The primers used for the RT-qPCR are listed in the Table 1, including their sequences and efficiencies. As targets in the rumen papilla epithelium, genes involved in the pro-inflammatory response [Toll-like receptor 4 (*TLR4*); Cluster differential 14 molecule (*CD14*); Myeloid differentiation factor 88 (*MyD88*); Nuclear factor kappa-B (*NF-*κ*B*); Interleukin 1-beta (*IL-1*β), 6 (*IL-6*), and 10 (*IL-10*); Interferon-gamma (*IFN*γ); Tumor necrosis factor-alpha (*TNF*α)], barrier function complex [Claudin 1, 2, 4, and 7 (*CLDN11*, *CLDN2*, *CLDN4*, *CLDN7*); Corneodesmosin (*CDSN*); Desmoglein 1 (*DSG1*); Occludins (*OCLN*); Zona occludens 1 (*ZO1*)], cellular nutrient transport [Monocarboxylate transporter, isoforms 1, 2, and 4 (*MCT1*, *MCT2*, *MCT4*)], cellular pH regulation [Anion exchange protein 2 (*AE2*); ATPase sodium/potassium transporting subunit alpha 1 (*ATP1A1*); Sodium/hydrogen exchanger isoform 1, 2, and 3 (*NHE1*, *NHE2*, *NHE3*); Down-regulated in adenoma (*DRA*), and cellular metabolism [3-hydroxybutyrate dehydrogenase 1 and 2 (*BDH1*, *BDH2*)]; 3-hydroxy-3-methylglutaryl CoA synthase 1 and 2 (*HMGCS1*, *HMGCS2*]) were chosen. As reference genes beta actin (*ACTB*), glyceraldehyde−3−phosphate dehydrogenase (*GAPDH*), hypoxanthine phosphoribosyltransferase 1 (*HPRT1*), ornithine decarboxylase antizyme 1 (*OAZ1*), and tyrosine 3-monooxygenase/tryptophan 5-monooxygenase activation protein zeta (*YWHAZ*) were considered. The amplicons of all primers were verified with PrimerBLAST^5^, dissociation curves were used to test for efficiency and specificity (Neubauer et al., 2019; Petri et al., 2019) and an additional dissociation stage was performed to verify the presence of a single PCR product. All reactions were run in duplicate and repeated when the cycle difference was more than 0.5 cycles between duplicates.

**TABLE 1:** Gene symbols, common names, primer sequences, and PCR efficiency for *Bos taurus* genes used in this manuscript for RT-qPCR.

**Official gene symbol**	**Common gene name^1^**	**Forward (F) and Reverse (R) Primer Sequence (5′–3′)**	**Correlation *R*^2^**	**PCR efficiency**	**References**
*BDH1*		F GACTGCCACCACTCCCTACAC	0.999	98.84	Oba et al.,2015
		R TCCGCAGCCACCAGTAGTAGT			
*CD14*		F ATCCACAGTCCAGCCGACAA	0.998	90.07	Neubauer et al., 2019
		R CAGCAGCAGCAGCAGGTAGG			
*CLDN1*		F CACAGCATGGTATGGCAATAGAA	0.998	99.02	Petri et al., 2018
		R CAGCAGCCCAGCCAATGA			
*CLDN4*		F GTGTTTGGCGTGCTGTTGTC	0.999	98.7	Petri et al., 2018
		R GGCCTTGGAGCTCTCATCAT			
*SLC26A3*	*DRA*	F TGCACAAAGGGCCAAGAAA	0.999	99.013	Oba et al., 2015
		R GCTGGCAACCAAGATGCTATG			
*DSG1*		F AGACAGAGAGCAATATGGCCAGT	0.998	92.48	Steele et al., 2012
		R TTCACACTCTGCTGACATACCATCT			
*HMGCS1*		F GCTCCGAGAGGATACTCATCAC	0.999	98.74	Neubauer et al., 2019
		R CGCCGAGCGTAAGTTCTTCT			
*IL-10*		F TTAAGGGTTACCTGGGTTGC	0.987	87.3	This manuscript
		R GCCTGTGGCATCACCTCTTC			
*IL-1*β		F CATGTGTGCTGAAGGCTCTC	0.989	108.3	This manuscript
		R GATACCCAAGGCCACAGGAA			
*IL-6*		F CCAATCTGGGTTCAATCAGG	0.979	82	This manuscript
		R CAGGATCTGGATCAGTGTTCTG			
*IFN-*γ		F GCAGCTCTGAGAAACTGGAGGA	0.982	83.1	This manuscript
		R ATGGCTTTGCGCTGGATCT			
*TNF*α		F CAAGTAACAAGCCGGTAGCC	0.998	77.2	This manuscript
		R AGATGAGGTAAAGCCCGTCA			
*MyD88*		F CGACGACGTGCTGATGGA	0.998	105.3	Neubauer et al., 2019
		R CCTGCTGCTGCTTCAGAATATAC			
*NF-*κ*B*		F ATACGTCGGCCGTGTCTAT	0.994	104	Jin et al., 2016
		R GGAACTGTGATCCGTGTAG			
*SLC16A1*	*MCT1*	F CATCATGTTGGCTGTCATGTATGG	0.998	90.47	Metzler-Zebeli et al., 2013
		R TCCTGCACAGTGTTACAGAAGGA			
*SLC16A3*	*MCT4*	F CTCACCACAGGGGTCCTTAC	0.998	103.35	Metzler-Zebeli et al., 2013
		R AAGTAGCGGTTGAGCATGATGA			
*SLC26A3*	*DRA*	F TGCACAAAGGGCCAAGAAA	0.999	99.01	Oba et al., 2015
		R GCTGGCAACCAAGATGCTATG			
*SLC9A1*	*NHE1*	F GAAAGACAAGCTCAACCGGTTT	0.998	92.1	Oba et al., 2015
		R GGAGCGCTCACCGGCTAT			
*TJP1*	*ZO1*	F AGCTCGGTGAACACGACAGA	0.997	95.73	Neubauer et al., 2019
		R TAGTACTCCTCATCCTCCTCGG			
*TLR4*		F GGTTTCCACAAAAGCCGTAA	0.984	92.6	Petri et al., 2018
		R AGGACGATGAAGATGATGCC			
*ACTB*^2^		F CGTGAGAAGATGACCCAGATCA	0.999	90.17	Steele et al., 2012
		R TCACCGGAGTCCATCACGAT			
*GAPDH*^2^		F TGGAAAGGCCATCACCATCT	0.999	90.95	Steele et al., 2012
		R CCCACTTGATGTTGGCAG			
*HPRT1*^2^		F TTGTATACCCAATCATTATGCTGAG	0.999	95.52	Petri et al., 2018
		R ACCCATCTCCTTCATCACATCT			
*OAZ1*^2^		F CACAAGAACCGTGATGATCGA	0.998	108.16	Petri et al., 2018
		R TCTCACAATCTCAAAGCCCAAA			
*YWHAZ*^2^		F TGAAAGGAGACTACTACCGCTACTTG	0.997	93.77	Petri et al., 2018
		R GCTGTGACTGGTCCACAATCC			

*The annealing temperature of 60°C was used for all primers. ^1^Given if the common gene name is differing from the official gene symbol. ^2^Considered as reference genes in this manuscript.*

NormFinder (Andersen et al., 2004) and BestKeeper (Pfaffl et al., 2004) were used to select the three best fitting genes from the five analyzed reference genes based on stable expression throughout the samples. *HPRT1*, *OAZ1*, and *YWHAZ* were found to be the most stable reference genes and their geometric mean was used to calculate the relative quantities of the target genes. The mRNA concentration of the target genes relative to the concentration of the reference genes was obtained by calculating the difference of the quantification cycle (Cq) between the target genes and the geometric mean of the three best fit reference genes for each sample. The average of the resulting delta cycle threshold ΔCq from the baseline feeding was used as reference value to calculate the ΔΔCq (Schmittgen and Livak, 2008). To calculate the relative expression for the target genes, the following formula was used: relative expression = 2^–ΔΔ*Cq*^ (Petri et al., 2018).

### Statistical Analyses

Statistical analyses for taxonomy and gene expression were performed using SAS (version 9.2, SAS, Inst. Inc., Cary, NC, United States). Overall, the treatments and blocking variables did not interact, as assumed for the Latin square design. Data were checked for normality and variance homogeneity prior to further statistical analysis. All statistical models were performed with the feeding phase (Baseline, SARAI, SARAII) and treatment (CON, PHY, AY) considered as fixed effects. Interactions between feeding phase and treatment were also tested. Cow nested within square and the experimental period were considered as a random effect. If no effects of interaction were found, baseline samples were removed and treatment effects were tested using the Dunnett-Hsu adjusted *P*-value for PHY and AY compared to CON, these effects are only given for feeding phases SARAI and SARAII because feed additives were only administered during those phases. Comparisons among the least square means were performed with pdiff option and considered as significant with *P* < 0.05 and as a trend with 0.05 ≤ *P* ≤ 0.10. Correlation analysis was performed using the ProcCorr procedure of SAS. According to Hinkle et al. (2003), the r was interpreted as follows: 0.00 to 0.30 as negligible; 0.30 to 0.50 as low; 0.50 to 0.70 as moderate; 0.70 to 0.90 as high, and 0.90 to 1.00 as substantial.

## Results

As part of a larger study, we have previously reported the effects of phytogenics and autolyzed yeast products on the rumen fermentation, ruminal pH and rumination activity (Kröger et al., 2017), rumen digesta- and liquid-associated microbes and fermentation products (Neubauer et al., 2018), and the role of feed additives on ruminal biogenic amines, LPS, and blood metabolome (Humer et al., 2018) under intermittent high grain feeding. Results of biogenic amines and LPS were used in this study to establish correlations with rumen microbiota community and host gene expression.

### Diversity of the Epithelial Microbiota

Non-parametric measures of alpha diversity were calculated for animals fed SARA diets and are shown in Supplementary Figure 2. For the diversity and richness indices of Shannon, Simpson, Chao1 and the total number of observed OTUs, no significant difference was found based on the supplementation of either AY or PHY in comparison to the CON. However, epithelial microbial diversity between feeding phases showed an increase in the Shannon index in SARA diets (4.46 and 4.50 in SARAI and SARAII, respectively) from an average of 4.26 in the Baseline (*P* = 0.04). Shannon index also tended to show an interaction between treatment and feeding regime (*P* = 0.09). In the AY treatment group Shannon index diversity was highest in SARAI (4.54), whereas in the CON and PHY groups, the highest index was found in SARAII (4.57 and 4.60, respectively).

### Microbial Composition

A total of 1150 OTUs were identified from 817,138 sequences in 72 samples with an average of 9,948 sequences per sample. This translated into 15 phyla representing 58 genera based on the SILVA database (v128). The relative abundance for all epithelial samples was dominated by *Actinobacteria* (5.9–9.3%), *Firmicutes* (43.3–46.1%), and *Proteobacteria* (41.5–46.7%). At the phylum level only *Chloroflexi* (*P* = 0.02), and GN02 (*P* = 0.02) were increased by the addition of PHY (Table 2). Analysis of the treatment × feeding phase interaction showed no significant effects at the phyla level. When treatment means were compared to CON, the supplementation of PHY increased the relative abundance of *Chloroflexi* by 1.67-fold (Dunnett-Hsu adjusted *P* = 0.01) and GN02 by 12.5-fold (Dunnett-Hsu adjusted *P* = 0.03).

**TABLE 2:** Relative abundance of epithelial microbiota during SARAI and SARAII identified to 97% at the phyla level of taxonomic classification using the RDP SILVA database (v128).

	**Treatment^1^**				***P-*value**
**Phyla**	**CON**	**PHY**	**AY**	**SEM**	**Additive**
*Actinobacteria*	11.3	10.0	7.1	1.24	0.62
*Bacteroidetes*	1.73	1.95	0.80	0.353	0.12
*Chloroflexi*	0.006^a^	0.049^b^	0.021^a^	0.0127	0.02
*Elusimicrobia*	0.040	0.032	0.022	0.0051	0.65
*Euryarchaeota*	0.167	0.232	0.145	0.0259	0.38
*Firmicutes*	50.1	45.1	50.2	1.66	0.39
GN02	0.002^a^	0.031^b^	0.005^a^	0.0090	0.02
*Planctomycetes*	0.014	0.034	0.008	0.0078	0.08
*Proteobacteria*	35.5	41.1	40.5	1.77	0.48
*Spirochaetes*	0.008	0.012	0.002	0.0030	0.57
*Synergistetes*	0.64	0.92	0.68	0.086	0.30
*Tenericutes*	0.09	0.08	0.29	0.067	0.10
TM7	0.22	0.22	0.15	0.023	0.57
*Verrucomicrobia*	0.017	0.015	0.001	0.0051	0.54
WPS2	0.000	0.000	0.006	0.0019	0.39

*SEM, standard error of the mean. *P*-value additive is based on the fixed effects ANOVA model using the Tukey–Kramer method. ^1^Treatment is the additive that was added to SARA diets; CON: control, no supplementation; PHY: phytogenic product; AY: autolyzed yeast. ^*a,b*^Differing superscripts in the same row indicate significant variation in comparison to the control group based on Dunnett-Hsu analysis.*

**FIGURE 1:**
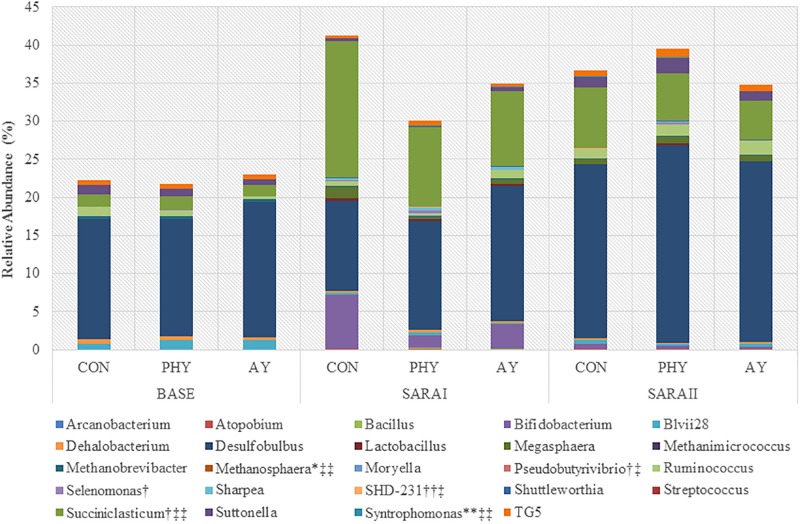
Relative abundance (%) of genera significantly (*P* ≤ 0.05) affected by feeding phase, additive treatment or an interaction of feeding phase with treatment. ^∗^Indicates a trend toward significant interaction, ^∗∗^Indicates a significant interaction. †Indicates a trend toward significant effect of treatment, †† Indicates a significant effect of treatment. ‡ Indicates a trend toward significant feeding phase ‡‡ Indicates a significant effect of feeding phase. Genera with no label have only a significant effect of feeding phase. Genera not including in graph that only had a tendency toward significant effect of feeding phase include: *Actinomyces*, *Phenylobacterium*, *Pyramidobacter*, *Ruminobacter*, and *Sphingomonas*.

Coverage of the rumen ecosystem at the genera level was on average 49% of the total sequences found, resulting in identification of 58 unique genera (Supplementary Table 1). At the phylum level only *Chloroflexi* (*P* = 0.02), and GN02 (*P* = 0.02) were increased by the addition of PHY (Table 2). In contrast, supplementation with AY showed a trend toward decreased *Succiniclasticum* when compared to CON (Figure 1). Dunnett-Hsu adjusted analysis of each additive when compared to the control showed trends toward decreases in *Bifidobacterium* (*P* = 0.08), as well as an increase in SHD-231 (*P* = 0.07) when PHY was supplemented. In contrast, only *Succiniclasticum* was found to decrease with AY supplementation compared to CON (*P* = 0.06). Analysis of the treatment × feeding phase interaction showed no significant effects at the genera level.

Analysis of the identified OTUs accounting for more than 0.15% of the total population of sequences was unable to accurately identify to the species level of taxonomy (Supplementary Table 2). However, one OTU identified as *Succiniclasticum*-like (97%) with a relative abundance of 2.54% was found to be significantly decreased by the supplementation with both PHY and AY. Analysis of the treatment × feeding phase interaction showed 4 OTUs with a significant interaction (OTU 7, 22, 33, and 39), all of which belong to the order Clostridiales but could not be identified at the genera level.

### Biomarker Genera Within the Epithelial Microbial Community

**FIGURE 2:**
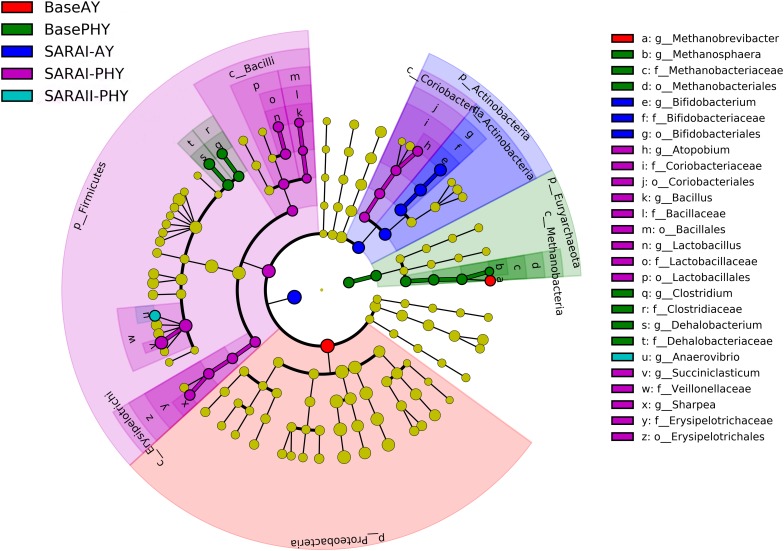
Linear discriminant analysis effect size (LEfSe) analysis to determine biomarker genera within the rumen microbial community of animals receiving either autolyzed yeast (AY) or a phytogenic compound (PHY) under Baseline 100% forage feeding or one of two SARA challenges of either 7 days (SARAI) or 14 days (SARAII) with a week of Baseline feeding in-between. Letter provided before the microbial taxa related to the taxonomic level (p_: phyla; c_: class; o_: order; f_: family; g_: genera).

Using the all sequencing data, LEfSe was performed in order to identify key bacteria groups related to both diet and feed additive using all microbial data. When data were compared using LEfSe without the effect of feeding, no significant differences were found for PHY and AY. However, with the added parameter of feeding phase, a large number of biomarker groups were identified (Figure 2). In the Baseline feeding period, the *Methanobrevibacter* genus was identified as a biomarker species for the AY treatment. Whereas in the PHY treatment, *Methanosphaera, Clostridium*, and *Dehalobacterium* were the biomarker genera identified. In the first SARA challenge, members of the genera *Bifidobacterium* were identified as key to AY, whereas, *Atopobium*, *Bacillus*, *Lactobacillus, Succiniclasticum*, and *Sharpea* were biomarker groups for the PHY group. During SARAII, only the PHY group had a biomarker group at the genera level, *Anaerovibrio*.

### mRNA Expression of Host Genes

Gene expression targets were identified in order to assess the general impact of feed additives on the host tissue pH-regulation mechanisms, cellular transport and metabolism, barrier function, and inflammation pathways (Table 3). Gene expression of *DRA* was increased with the addition of AY in the diet compared to CON (*P* = 0.02). Expression of *NHE1* increased with AY supplementation (*P* = 0.05). Relative expression of gene target *BDH1* was significantly increased with the supplementation of AY. Expression of cellular transport gene target *MCT1* and *MCT4* were significantly increased with the addition of AY in the diet. Changes in gene expression related to cellular metabolism included a treatment effect for *HMGCS2* with a significant increase in AY supplemented animals compared to CON and a 32% increase in *BDH1* expression with AY supplementation (*P* = 0.002). Three barrier function associated genes showed a positive effect of feed additive on expression, including *CLDN1*, *CLDN4*, and *ZO1*. Analysis with Dunnett-Hsu adjustment also identified a significant increase for all three genes with the supplementation of AY compared to the CON diet.

**TABLE 3:** Relative transcript (ΔΔCq) abundance for all gene targets based on effect of additive supplementation.

	**Treatment^1^**				
	**CON**	**PHY**	**AY**	**SEM**	***P*-value additive**
**pH Regulation**					
AE2	0.03	0.03	0.04	0.001	0.85
ATP1A1	1.15	0.97	1.16	0.062	0.30
DRA	3.15^a^	3.65^a^	5.66^b^	0.766	0.02
NHE1	0.08^a^	0.08^a^	0.10^b^	0.008	0.05
NHE2	0.50	0.56	0.51	0.019	0.58
NHE3	0.26	0.30	0.23	0.020	0.40
PAT1	0.01	0.01	0.01	0.001	0.44
**Nutrient transport/cellular metabolism**					
BDH1	0.92^a^	1.19^a^	1.55^b^	0.180	0.003
BDH2	0.11	0.10	0.10	0.003	0.70
HMGCS1	0.62	0.43	0.64	0.068	0.32
HMGCS2	4.29	3.55	5.24	0.487	0.04
MCT1	1.82^a^	2.39^a^	3.23^b^	0.410	0.003
MCT2	0.002	0.002	0.002	0.0001	0.77
MCT4	0.002^a^	0.002^a^	0.003^b^	0.0003	0.03
**Inflammation**					
CD14	0.08	0.08	0.07	0.004	0.46
IL10	0.0001	0.0002	0.0001	0.00002	0.28
IL1β	0.003	0.004	0.003	0.000	0.50
IL6	0.0004	0.0009	0.0003	0.00019	0.47
INFγ	0.0003	0.0003	0.0003	0.00001	0.84
MyD88	0.08	0.11	0.11	0.009	0.15
NFκB	0.06^a^	0.09^b^	0.10^b^	0.012	0.02
TLR4	0.006	0.008	0.006	0.0008	0.11
TNFα	0.003	0.003	0.002	0.0002	0.39
**Barrier function**					
CDSN	0.001	0.001	0.0004	0.00016	0.38
CLDN1	1.17^a^	1.33^a^	1.78^b^	0.182	0.02
CLDN2	0.00001	0.00001	0.00002	0.000001	0.59
CLDN4	1.46^a^	1.39^a^	2.38^b^	0.317	0.001
CLDN7	0.02	0.02	0.02	0.002	0.32
DSG1	0.16	0.11	0.17	0.019	0.40
OCLN	0.19	0.20	0.20	0.002	0.97
ZO1	0.43^a^	0.47^a^	0.63^b^	0.059	0.01

*SEM, standard error of the mean. *P*-value additive is based on the fixed effects ANOVA model using the Tukey–Kramer method. ^1^Treatment is the additive that was added to SARA diets; CON: control, no supplementation; PHY: phytogenic product; AY: autolyzed yeast. ^*a,b*^Differing superscripts in the same row indicate significant variation in comparison to the control group based on Dunnett-Hsu analysis.*

### Correlation Analysis for Rumen Epithelial Ecology

**FIGURE 3:**
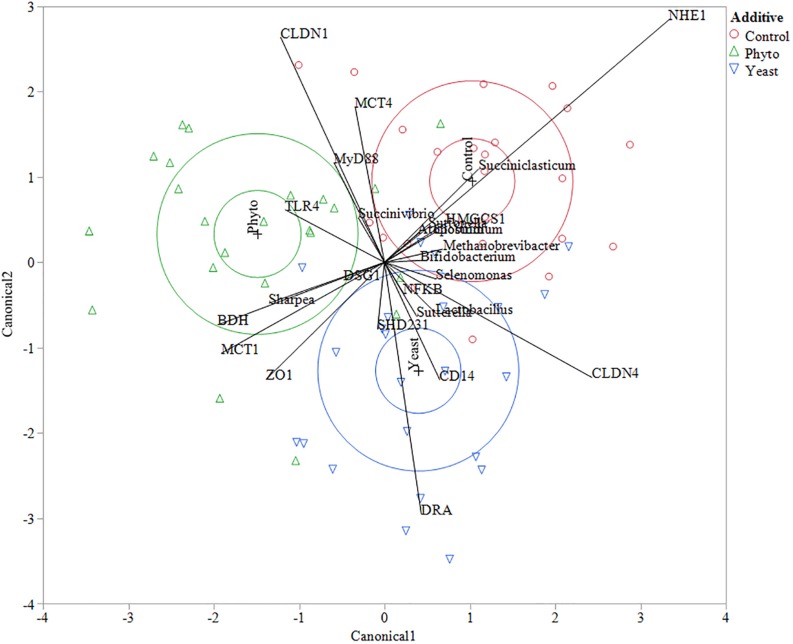
Canonical correlation analysis between genes and biomarker species determined using LEfSe analysis. Control treatment samples are indicated by red circles, phytogenic treatment samples by green triangles and autolyzed yeast samples are indicated by blue down facing triangles. The larger circles in each treatment color represent the 50% contours representing the region in the space where 50% of the observations for each group should fall and the 95% confidence ellipses which show the 95% ellipsoids for the mean of each group. The eigenvalues 1 and 2 represent 55.96 and 44.04% of the variation, respectively. Wilks’ Lambda test gave a *F*-test value of 0.0119.

Multivariate constrained ordination (CCA) was performed using the biomarker genera indicated from the LEfSE analysis and all of the tested gene expression targets with reference to the microbial community composition of each individual sample. Clustering based on feed additive was determined using canonical likelihood (Figure 3) with the first eigenvalue corresponding to 56% of the variation in the samples (*P* = 0.01). Interestingly, a number of correlations between specific genes and biomarker species with reference to feed additive were seen. Animals fed the CON showed a strong positive correlation to *NHE1* expression and *Succiniclasticum* abundance, as well as a negative correlation to *MCT1* and *ZO1* expression. Autolyzed yeast supplementation correlated positively with *CD14* and *DRA* and the abundance of *Lactobacillus* but negatively correlated with *CLDN1*, *MyD88*, *MCT4* and genus *Succinivibrio*. Supplementation with PHY showed positive correlations to *TLR4* and a negative correlation to *CLDN4.*

Pearson correlation analysis was performed to assess the relationship between ruminal biogenic amines on the entire rumen epithelial community and host gene expression under high grain feeding. Both cadaverine and tyramine were significantly associated with an increased abundance of *Oscillospira* (*r* = 0.78 and *r* = 0.79, respectively; *P* ≤ 0.001), *Coprococcus* (*r* = 0.83 and *r* = 0.85, respectively; *P* ≤ 0.001), and *Anerostipes* (*r* = 0.83 and *r* = 0.84, respectively; *P* ≤ 0.001). *Methanobrevibacter* was also positively correlated to levels of histamine in the plasma (*r* = 0.81; *P* = 0.001). Comparatively, gene expression showed only moderate correlations to biogenic amines and LPS. Negative associations included the level of isopropylamine and the gene expression for barrier function gene *CLDN1* (*r* = −0.64; *P* = 0.008), pH regulation gene targets *NHE1* (*r* = −0.56; *P* = 0.02), *NHE2* (*r* = −0.65; *P* = 0.01), *AE2* (*r* = −0.61; *P* = 0.01), and epithelial metabolic function gene *BDH2* (*r* = −0.61; *P* = 0.01). Other negative correlations included between putrescine and *CLDN4* (*r* = −0.52; *P* = 0.04) as well as ethanolamine and *MCT4* (*r* = −0.59; *P* = 0.02). Positive correlations with inflammation gene target *IL-6* included pyrrolidine (*r* = 0.58; *P* = 0.02) and LPS (*r* = 0.57; *P* = 0.02).

**FIGURE 4:**
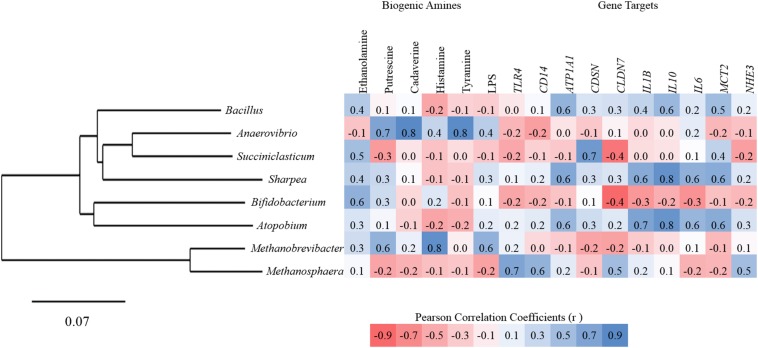
Pearson correlation between biomarker genera, rumen papillae gene expression, biogenic amines, and endotoxins for all animals.

Additional Pearson correlation analysis was performed using SAS (version 9.2) to assess the direct correlations between treatment specific biomarker genera from the high grain diets and gene expression for all targets, ruminal concentrations of biogenic amines and LPS. *Anaerovibrio* showed the strongest positive correlations to biogenic amines including putrescine (*r* = 0.67), cadaverine (*r* = 0.84), and tyramine (*r* = 0.83). *Succiniclasticum* and *Bifidobacterium* were both moderately positively correlated to ethanolamine (Figure 4). Significant correlations between high grain diet biomarker genera and gene targets were all positive in association but only moderate (0.5 ≤ *r* ≤ 0.7) with *Atopobium* and *Sharpea* both correlating to *ATP1A1*, *IL-1*β, *IL-10*, *IL-6*, and *MCT2*, and *Succiniclasticum* correlated with *CSDN* (Figure 4).

## Discussion

This study was conducted as part of a larger experiment looking at feed additives and detailed information about the experimental design and feeding, as well as the results for ruminal pH, chewing activity, ruminal microbiome, plasma associated metabolome, and LPS, have been reported in our companion papers (Kröger et al., 2017; Humer et al., 2018; Neubauer et al., 2018). The aim of this study was to determine the effect of supplementation of PHY or AY feed additives under repeated bouts of grain-induced SARA on rumen epithelial microbial populations, epithelial gene expression, and their interaction with regards to barrier function, and pro-inflammatory signaling.

### Role of AY

Numerous studies have reported positive effects of yeast products on rumen fermentation and microbial activity, either through the provision of limiting substrates (Callaway and Martin, 1997; Mao et al., 2013) or through inhibition Gram-negative bacteria (Ganner and Schatzmayr, 2012). In the rumen, Gram-negative bacteria are the source of the rumen endotoxin, a pro-inflammatory molecule that has been attributed to several symptoms of SARA, and is known to increase in luminal concentration under high-grain feeding (Plaizier et al., 2012). Gram-negative bacteria release the endotoxin during exponential growth phase and especially during lysis in response to decreased pH (Plaizier et al., 2012); therefore, the inhibition of Gram-negative bacteria during a SARA challenge could potentially reduce the risk of the endotoxin-induced inflammation. In the current study, the rumen epithelial population consisted of predominantly Gram-negative phyla in the baseline feeding (56%) but significantly less Gram-negative phyla in the two bouts of SARA (30 and 47% in SARAI and SARAII, respectively). When analyzing the impact of AY supplementation, effects were only seen at the OTU level with a significant decrease in the Gram-negative *Succiniclasticum*-like OTU4 accounting for 2.5% of the relative epithelial abundance. *Succiniclasticum* spp. are associated with the feeding of high grain diets, and are a functionally important rumen genera that ferments succinate to propionate during the fermentation of carbohydrates (van Gylswyk, 1995). However, the role of *Succiniclasticum* spp. in the production of endotoxins remains unknown. In the companion paper, Neubauer et al. (2018), a shift in the ratio of Gram-negative to Gram-positive bacteria was also seen in the rumen digesta and rumen liquid microbial populations, with a notable decrease in the *Succiniclasticum* spp. in association with the supplementation AY. This information taken together with the epithelial microbiota data from the current study, shows an impact of AY on the Gram-negative bacteria in all niches (solid, liquid, and epithelial) of the rumen bacterial ecosystem.

Identifying the most biologically informative components differentiating two or more treatments can be challenging in rumen microbial datasets due to inherent sequencing bias, complex rumen interactions, and high inter-animal variability. From the mixed model analysis of phylogeny, no effect of treatment was found for *Bifidobacterium*; however, it was the only bacterial genera determined as a biomarker genus for AY supplementation. Previous research has shown that the addition of yeast cell wall components to the rumen can act as a binding agent for Gram-negative bacteria and result in an increase in beneficial bacteria such as *Lactobacilli* and *Bifidobacteria* (Ganner and Schatzmayr, 2012).

From an animal health and immunity perspective, yeast cell wall components from *Saccharomyces cerevisiae* have been reported to promote the synthesis and release of the pro-inflammatory cytokines and are involved in the release of other cytokines such as *IL-1*β, *IL*-2, and *IL*-6 (Medzhitov and Janeway, 2000; Brown, 2005). A subacute inflammatory response could contribute to chronic and progressive changes in the tissue function (Bradford et al., 2015). However, in the current study there was no effect of feed additive on the pro-inflammatory cytokines. The supplementation of AY in the current study resulted in an increase in the relative expression of pH regulation gene targets (*DRA* and *NHE1*), nutrient transport and cell metabolism targets (*BDH1*, *HMGCS2*, *MCT1*, and *MCT4*). However, Kröger et al. (2017) reported that supplementation with AY increased DMI by 20% and increased eating time compared with CON, resulting in a trend toward increased ruminal pH. Increased DMI and eating time are in agreement with our finding that AY supplementation increased the relative expression of nutrient transporters and cell metabolism gene targets in the rumen epithelium. The basis of SARA is the rapid production of SCFA in the rumen, often resulting in an accumulation over extended periods of time after an eating event (Zebeli et al., 2008). An increase in nutrient transporter gene expression could indicate rumen epithelial adaptation, and a subsequent increase in SCFA removal from the rumen. The increase in pH regulation gene expression and cell metabolism gene target expression, would support the finding of increased transport of nutrients into the cell. Kröger et al. (2017) found an increase in DMI but no decrease in pH under AY supplementation. This would indicate an increase in fermentable substrate but associated with no reduction in pH. These findings together support the hypothesis that AY supplementation increased epithelial uptake of SCFA and cellular metabolism.

In the current study, supplementation with AY also resulted in an increase in the relative expression of barrier function targets (*CLDN1, CLDN4*, and *ZO1*) as well as *NF-K B.* Increases in barrier function gene targets have also been previously associated with increases in DMI (Petri et al., 2018), and in association with lower pH (McCann et al., 2016) in SARA induction. However, the exact mechanism regarding the impact of ruminal pH on barrier proteins remains unclear. In the current study, the cattle were subjected to multiple bouts of SARA through a high grain feeding challenge, which has been previously shown to impact the host epithelial gene expression and recovery of epithelial tissue (Petri et al., 2019). Zanello et al. (2011) reported that yeast downregulated the expression of genes involved in inflammation and recruitment and activation of immune cells in intestinal epithelial cells, specifically *IL-6*. Supplementation with AY in the current study did not impact any of the measured inflammatory cytokine expression but did increase the expression of *NF-*κ*B*. Despite minor increases in the gene expression of *NF-*κ*B*, no inflammation was found in the animals under the experimental conditions. Gene expression changes are not indicative of protein translation, and therefore further studies looking at localized inflammation should consider proteomic analysis of the rumen epithelium. Furthermore, no effect in the expression of *TLR4* supports previous research that has shown the adaptations in TLR4 expression occur within 24 h of a single bout of SARA (Liu et al., 2015; McCann et al., 2016). The role of TLR4 in the epithelium is not only to initiate the inflammatory response by binding pathogen-associated molecules (Akira and Takeda, 2004), but also to aid in cell survival and tissue repair through the *NF-*κ*B* mediated suppression of apoptosis (Li et al., 2010).

### Role of PHY

Despite minimal changes in the diversity, changes in the phylogeny were seen at all taxonomy levels. Normalized relative abundances showed numerical increases compared to CON in *Actinobacteria*, *Bacteroidetes*, *Firmicutes* and *Proteobacteria* when PHY are given, regardless of dietary regime. This indicates an alteration in the rumen environment possibly by providing more favorable conditions for microbial growth based on selective pressure on microbial populations through impacting cell wall integrity (Neubauer et al., 2018). In the current study only two phyla, *Chloroflexi* (Gram-negative) and *Planctomycetes* (no gram stain), were found to be significantly higher when PHY was supplemented. The phylum *Chloroflexi*, previously called green non-sulfur bacteria (GNS), is found in many environments (Garrity and Holt, 2001) and within this phyla there are approximately 30 named species in over 20 genera. Of these, class *Anaerolineae* is the only class known to have host-associated species (Camanocha and Dewhirst, 2014). At the genera level, SHD-231 (class *Anaerolineae*) doubled in relative abundance associated with PHY supplementation. Unfortunately, the metabolic role of these groups is not understood in the rumen and especially with reference to the rumen epithelium. While extensive research has been done in the last decade with regard to determining the key taxa associated with the rumen epithelial community, still very few have been cultured and therefore, very little information is known about their metabolic role and their impact on the host.

In comparison, genera *Selenomonas* is one of the original rumen bacteria grown and analyzed using classical microbiology (Hungate, 1966). In the current study, *Selenomonas* tended to be increased in relative abundance under PHY supplementation. The most common ruminal species is *S*. *ruminantium*, a well-known Gram-positive rumen microbe, which is reduced during lactic-acid accumulation (Meissner et al., 2010). In our companion study, Neubauer et al. (2018) reported no effect of phytogenic and autolyzed yeast feed additives on total lactate in the rumen. This is to be expected under SARA conditions where lactate does not accumulate in comparison with lactic acidosis. However, Dinsdale et al. (1980) found *Selenomonas* to also be a critical part of the rumen epithelial microbial community, contributing to the breakdown of ruptured epithelial cells under low pH conditions. Therefore, a shift toward *Selenomonas* populations under PHY supplementation, especially under low pH where lactic acid accumulates and epithelial cell turnover is high, could provide an advantage to the host in mitigating acidosis severity and by increasing energy supply to the host in the form of propionate.

The use of LEfSe was performed in order to attempt to identify key bacteria groups related to both diet and feed additive. This method of biomarker species prediction has been used in human gastrointestinal research to assess host factors such as lifestyle and disease (Segata et al., 2011). Five genera were determined as biomarker genera for PHY in SARAI including *Atopobium, Bacillus, Lactobacillus, Succiniclasticum*, and *Sharpea.* Only *Succiniclasticum* showed a trend toward decreasing with PHY supplementation, all other genera were did not have treatment related effects. In SARAII only one biomarker genus was found in association with PHY, *Anaerovibrio*, which also showed a numerical increase in the PHY treatment group but no statistical significance. Differences between the statistical analysis and the LEfSe analysis can be expected based on the reduced database used in LEfSe classification. This of course removes some of the rarer OTUs and can reduce the amount of information about the rumen ecosystem. However, the genetic diversity in the rumen microbiota is extensive and complex, making an overview analysis of this ecosystem difficult without first restricting the dataset. Both molecular and bioinformatic tools include inherent biases based on limitations within the methodology. However, it is important that we use multiple techniques to better assess the changes within the rumen ecosystem under various feeding conditions. The variation in the statistically significant populations found in sequencing compared to those genera found to be biomarker taxa for specific diet × treatment interactions was expected based on the reduced database bias. Therefore, it is important to put the results of such analysis within the context of the rumen environment at the time of sampling. In this regard, we performed a Pearson correlation analysis with ruminal concentrations of biogenic amines and LPS (Humer et al., 2018). Biogenic amines are naturally occurring nitrogenous compounds synthesized by plants, animals and microorganisms, mainly through the decarboxylation of amino acids; however, ingestion can provoke serious toxicological reactions (Del Rio et al., 2019). In the current study, the highest correlation to biogenic amines was found between *Anaerovibrio* and the biogenic amines cadaverine and tyramine. Hirao et al. (2000) previously reported that spermidine and cadaverine are constituents of the cell wall peptidoglycan of rumen bacteria *Anaerovibrio lipolytica* and that these diamines are essential for both cell surface integrity and normal cell growth. In the present study, there was no correlation between the genera *Anaerovibrio* and the ruminal concentration of spermidine. Buňková et al. (2009) reported that the production of biogenic amines in bacteria seems to be strain-dependent rather than related to bacterial species or even genera. It could mean that there are other *Anaerovibrio* spp. besides lipolytica that colonize the rumen wall. The ruminal concentration of tyramine highly correlate with *Anaerovibrio* might also indicate that the level of biogenic amines is not associated with release from the cell wall of lyzed microbes but instead from the increased amino acid metabolism of the epithelial microbiota under specific conditions. Cadaverine is produced from the decarboxylation of lysine, and tyramine from tyrosine (Buňková et al., 2009). Previous research looking at the predicted metabolism (PICRUSt) of the rumen microbiota under acidosis conditions indicated a significant increase in the production of amino acid related enzymes and biosynthesis of tyrosine under two different SARA models (Petri et al., 2017). However, the metabolic pathways of rumen epithelial microbiota have yet to be researched under SARA conditions in the rumen. Since changes in biogenic amines can be seen under low pH conditions (Humer et al., 2018), without large increases in pathogenic bacteria (Neubauer et al., 2018), it can be speculated that a change in the metabolism of rumen epithelial microbiota, not the alteration in relative abundances of pathogenic microbiota, are the underlying cause for the production of biogenic amines and endotoxins. The mode of cytotoxic action putrescine and cadaverine is the initiation of cell necrosis (Del Rio et al., 2019). Histamine, a highly pro-inflammatory amine, has been previously speculated as the causative agent in rumen epithelial inflammation and during SARA due to its role in laminitis (Nocek, 1997). However, histamine showed no correlations to any of the biomarker genera, which was expected since no inflammation was seen in the animals during the current studyThe increase in the relative abundance of *Anaerovibrio* that was associated with an increase in toxic biogenic amines in SARAII may imply that despite the increase in ruminal pH in SARAII, the metabolic shift in epithelial microorganisms toward increased amino acid metabolism may be a long term metabolic strategy as it occurred after a longer period of SARA challenge.

Assessment of the effect of PHY supplementation on host gene expression only showed an increase in the relative abundance of *NF-*κ*B*. As previously mentioned, the increase in *NF-*κ*B* may provide a host benefit under low-pH conditions by reducing cell apoptosis (Li et al., 2010). However, correlation analysis showed moderate and high correlations between biomarker genera associated with PHY supplementation in SARAI and cytokine gene expression in the rumen papillae. Positive correlations between *Sharpea* and *Atopobium* with *IL-1*β, *IL-10* and *IL-6*, along with nutrient transporter *MCT2* would indicate a positive influence of PHY on biomarker genera and their impact on the host gene expression. *IL-10* is an anti-inflammatory and *IL-1*β inhibits *NF-*κ*B*. None of the biomarker genera had a correlation to *TLR4* expression in the host which would indicate that there are other forms of host-microbiota cross-talk which have yet to be elucidated in the rumen.

Analysis of sequencing data, including the biases of PCR, quality control parameters, database usage, and *post hoc* statistical analysis such as LEfSe can result in limitations to data interpretation. Despite optimization of sample preparation, DNA extraction, and method analysis, matrixes such as the epithelial tissue can still show large variation, which results in large reductions in datasets in an attempt to improve data quality. These limitations are important to recognize in attempts to interpret data; however, it is critical that the analysis is repeated in an attempt to improve our understanding of this complex microbial environment.

In our previous publications, PHY supplementation altered the relative abundance of a larger number of rumen digesta associated microbiota compared to the number of rumen epithelial microbiota changed in abundance as shown in the current study. There are also relatively low changes in gene expression compared to previous studies in regards to host epithelial gene expression (Petri et al., 2019). In general, the impact of feed additives on the rumen microbiota has been more extensively studied in the literature in comparison to the impact on the host epithelial gene expression and host-associated microbiota. This is a critical gap of knowledge when attempting to provide feed additives that will stabilize the rumen ecosystem from an overall microbial dysbiosis under SARA conditions, as the rumen epithelium is a significant component of that ecosystem. In addition, the complexity of the analysis should be considered for products that contain multiple components (i.e., oils, herbs, spices). Therefore, further research will require that we not only understand the physiology of rumen fermentation and digestion, but also the host-associated adaptations and the communication between these components in the form of various metabolites including biogenic amines through the integration of various types of ‘omics analysis, especially in response to dosed feed additives.

Ruminal dysbiosis continues to be an issue for the modern dairy industry due to the necessary feed management required in order for high producing animals to meet their energy requirements, resulting in low ruminal pH’s. The effectiveness of nutritional intervention strategies for the prevention of ruminal dysbiosis under modern feeding regimes remains unclear due to the complexity of the rumen ecosystem. However, the rumen epimural community, due to proximity, has the greatest opportunity to influence host gene expression with regards to barrier function, cell function and localized inflammation in high energy diets. The results of this study show that the AY and PHY products used in this study have different impacts on the rumen microbial community, and host gene expression. The addition of PHY tended to impact the rumen epithelial microbiota, whereas AY tended to impact more epithelial gene expression targets. The results showed that supplementing feed additives to high grain production diets can stabilize epithelial microbial community under low pH conditions of SARA, which then correlates to changes in the SARA associated host gene expression. The correlations between measured biogenic amines and rumen epithelial microbiota indicates that to improve our understanding of the rumen ecosystem, it is important to understand the relationships between diet and the production of metabolic substrates such as biogenic amines and endotoxins.

## Data Availability Statement

The datasets generated for this study can be found in the sequencing data were deposited into the European Nucleotide Archive (ENA) and can be accessed via accession numbers PRJEB33839 and PRJEB29866 for CON samples.

## Ethics Statement

The animal study was reviewed and approved by the Austrian National Authority for Law for Animal Experiments and the University of Veterinary Medicine Ethics Committee.

## Author Contributions

QZ and NR designed the experiments. VN, IK, and EH performed the experiments. RP analyzed and interpreted the data and drafted the manuscript. All authors read and approved the final version of the manuscript.

## Conflict of Interest

NR was employed by BIOMIN Holding GmbH and VN was employed by the non-profit research center FFoQSI GmbH. The remaining authors declare that the research was conducted in the absence of any commercial or financial relationships that could be construed as a potential conflict of interest.

## Footnotes

1 http://qiime.org/

2 http://qiime.org/tutorials/index.html

3 https://www.arb-silva.de

4 http://huttenhower.sph.harvard.edu/galaxy/

5 https://www.ncbi.nlm.nih.gov/tools/primer-blast/

## References

[B1] Akira S. Takeda K. (2004). Toll-like receptor signalling. *Nat. Rev. Immunol.* 4 499–511. 10.1038/nri1391 15229469

[B2] Andersen C. L. Jensen J. L. Ørntoft T. F. (2004). Normalization of real-time quantitative reverse transcription-pcr data: a model-based variance estimation approach to identify genes suited for normalization, applied to bladder and colon cancer data sets. *Cancer Res.* 64 5245–5250. 10.1158/0008-5472.can-04-0496 15289330

[B3] Bokulich N. A. Subramanian S. Faith J. J. Gevers D. Gordon J. I. Knight R. (2013). Quality-filtering vastly improves diversity estimates from Illumina amplicon sequencing. *Nat. Methods* 10 57–59. 10.1038/nmeth.2276 23202435 PMC3531572

[B4] Bradford B. J. Yuan K. Farney J. K. Mamedova L. K. Carpenter A. J. (2015). Invited review: inflammation during the transition to lactation: new adventures with an old flame. *J. Dairy Sci.* 98 6631–6650. 10.3168/jds.2015-9683 26210279

[B5] Brown G. D. (2005). Dectin-1: a signalling non-TLR pattern-recognition receptor. *Nat. Rev. Immunol.* 6 33–43. 10.1038/nri1745 16341139

[B6] Buňková L. Buňka F. Hlobilová M. Vaňátková Z. Nováková D. Dráb V. (2009). Tyramine production of technological important strains of *Lactobacillus*, *Lactococcus* and *Streptococcus*. *Eur. Food Res. Tech.* 229 533–538. 10.1007/s00217-009-1075-3 8005830

[B7] Callaway E. S. Martin S. A. (1997). Effects of a *Saccharomyces cerevisiae* culture on ruminal bacteria that utilize lactate and digest cellulose. *J. Dairy Sci.* 80 2035–2044. 10.3168/jds.s0022-0302(97)76148-4 9313145

[B8] Camanocha A. Dewhirst F. E. (2014). Host-associated bacterial taxa from Chlorobi, Chloroflexi, GN02, Synergistetes, SR1, TM7, and WPS-2 Phyla/candidate divisions. *J. Oral Microbiol.* 6:25468. 10.3402/jom.v6.25468 25317252 PMC4192840

[B9] Caporaso J. G. Kuczynski J. Stombaugh J. Bittinger K. Bushman F. D. Costello E. K. (2010). QIIME allows analysis of high-throughput community sequencing data. *Nat. Methods* 7 335–336. 10.1038/nmeth.f.303 20383131 PMC3156573

[B10] Chaucheyras-Durand F. Fonty G. (2002). Influence of a probiotic yeast (Saccharomyces cerevisiae CNCM I-1077) on microbial colonization and fermentations in the rumen of newborn lambs. *Microb. Ecol. Health Dis.* 14 30–36. 10.1080/089106002760002739

[B11] Del Rio B. Redruello B. Linares D. M. Ladero V. Ruas-Madiedo P. Fernandez M. (2019). The biogenic amines putrescine and cadaverine show in vitro cytotoxicity at concentrations that can be found in foods. *Sci. Rep.* 9:120. 10.1038/s41598-018-36239-w 30644398 PMC6333923

[B12] Dinsdale D. Cheng K. J. Wallace R. J. Goodlad R. A. (1980). Digestion of epithelial tissue of the rumen wall by adherent bacteria in infused and conventionally fed sheep. *Appl. Environ. Microbiol.* 39 1059–1066. 10.1128/aem.39.5.1059-1066.1980 6772103 PMC291475

[B13] Duffield T. Bagg R. N. (2000). Use of ionophores in lactating dairy cattle: a review. *Can. Vet. J.* 41 388–394. 10816832 PMC1476247

[B14] Edgar R. C. (2010). Search and clustering orders of magnitude faster than BLAST. *Bioinformatics* 26 2460–2461. 10.1093/bioinformatics/btq461 20709691

[B15] Flachowsky G. Lebzien P. (2012). Effects of phytogenic substances on rumen fermentation and methane emissions: a proposal for a research process. *Anim. Feed Sci. Technol.* 176 70–77. 10.1016/j.anifeedsci.2012.07.009

[B16] Ganner A. Schatzmayr G. (2012). Capability of yeast derivatives to adhere enteropathogenic bacteria and to modulate cells of the innate immune system. *Appl. Microbiol. Biotechnol.* 95 289–297. 10.1007/s00253-012-4140-y 22615053

[B17] Garrity G. M. Holt J. G. (2001). “Phylum BVI chloroblexi phy. nov,” in *Bergey’s Manual of Systematic Bacteriology*, 2nd Edn, eds Boon D. R. Castenholtz R. W. Garrity G. M. (New York, NY: Springer), 427–446. 10.1007/978-0-387-21609-6_23

[B18] Harrison G. A. Hemken R. W. Dawson K. A. Harmon R. J. Barker K. B. (1988). Influence of addition of yeast culture supplement to diets of lactating cows on ruminal fermentation and microbial populations. *J. Dairy Sci.* 71 2967–2975. 10.3168/jds.s0022-0302(88)79894-x 3230186

[B19] Hinkle D. E. Wiersma W. Jurs S. G. (2003). *Applied Statistics for the Behavioral Sciences.* Boston, MA: Houghton Mifflin College Division.

[B20] Hirao T. Sato M. Shirahata A. Kamio Y. (2000). Covalent linkage of polyamines to peptidoglycan in anaerovibrio lipolytica. *J. Bacteriol.* 182 1154–1157. 10.1128/jb.182.4.1154-1157.2000 10648544 PMC94394

[B21] Humer E. Kröger I. Neubauer V. Schedle K. Reisinger N. Zebeli Q. (2018). Supplementing phytogenic compounds or autolyzed yeast modulates ruminal biogenic amines and plasma metabolome in dry cows experiencing subacute ruminal acidosis. *J. Dairy Sci.* 101 9559–9574. 10.3168/jds.2018-14744 30031584

[B22] Hungate R. E. ed (1966). “Chapter II - the rumen bacteria,” in *The Rumen and its Microbes.* Cambridge, MA: Academic Press, 8–90. 10.1016/b978-1-4832-3308-6.50005-x

[B23] Jin D. Chang G. Zhang K. Guo J. Xu T. Shen X. (2016). Rumen-derived lipopolysaccharide enhances the expression of lingual antimicrobial peptide in mammary glands of dairy cows fed a high-concentrate diet. *BMC Vet. Res.* 12:128. 10.1186/s12917-016-0755-z 27350130 PMC4924273

[B24] Kleen J. L. Hooijer G. A. Rehage J. Noordhuizen J. P. T. M. (2003). Subacute ruminal acidosis (SARA): a review. *J. Vet. Med. A Physiol. Pathol. Clin. Med.* 50 406–414. 10.1046/j.1439-0442.2003.00569.x 14633219

[B25] Kröger I. Humer E. Neubauer V. Reisinger N. Aditya S. Zebeli Q. (2017). Modulation of chewing behavior and reticular pH in nonlactating cows challenged with concentrate-rich diets supplemented with phytogenic compounds and autolyzed yeast. *J. Dairy Sci.* 100 9702–9714. 10.3168/jds.2017-12755 28964521

[B26] Li X. Jiang S. Tapping R. I. (2010). Toll-like receptor signaling in cell proliferation and survival. *Cytokine* 49 1–9. 10.1016/j.cyto.2009.08.010 19775907 PMC2808458

[B27] Liu J. Bian G. Zhu W. Mao S. (2015). High-grain feeding causes strong shifts in ruminal epithelial bacterial community and expression of Toll-like receptor genes in goats. *Front. Micro.* 6:167. 10.3389/fmicb.2015.00167 25784904 PMC4345813

[B28] Mao H.-L. Mao H.-L. Wang J. K. Liu J. X. Yoon I. (2013). Effects of *Saccharomyces cerevisiae* fermentation product on in vitro fermentation and microbial communities of low-quality forages and mixed diets. *J. Anim. Sci.* 91 3291–3298. 10.2527/jas.2012-5851 23572258

[B29] McCann J. C. Luan S. Cardoso F. C. Derakhshani H. Khafipour E. Loor J. J. (2016). Induction of subacute ruminal acidosis affects the ruminal microbiome and epithelium. *Front. Microbiol.* 7:701. 10.3389/fmicb.2016.00701 27242724 PMC4870271

[B30] Medzhitov R. Janeway C. (2000). Innate immunity. *N. Engl. J. Med.* 343 338–344. 10.1016/S0952-7915(02)00019-5 10922424

[B31] Meissner H. Henning P. Horn C. Leeuw K.-J. Hagg F. Fouche G. (2010). Ruminal acidosis: a review with detailed reference to the controlling agent *Megasphaera elsdenii* NCIMB 41125 (Review). *South Afr. J. Anim. Sci.* 40:57275 10.4314/sajas.v40i2.57275

[B32] Metzler-Zebeli B. U. Hollmann M. Sabitzer S. Podstatzky-Lichtenstein L. Klein D. Zebeli Q. (2013). Epithelial response to high-grain diets involves alteration in nutrient transporters and Na+/K+-ATPase mRNA expression in rumen and colon of goats1. *J. Anim. Sci.* 91 4256–4266. 10.2527/jas.2012-5570 23825322

[B33] Navas-Molina J. A. Peralta-Sánchez J. M. González A. McMurdie P. J. Vázquez-Baeza Y. Xu Z. (2013). Advancing our understanding of the human microbiome using QIIME. *Microb. Metagen. Metatrans. Metaproteom.* 531 371–444. 10.1016/b978-0-12-407863-5.00019-8 24060131 PMC4517945

[B34] Neubauer V. Humer E. Mann E. Kröger I. Reisinger N. Wagner M. (2019). Effects of clay mineral supplementation on particle-associated and epimural microbiota, and gene expression in the rumen of cows fed high-concentrate diet. *Anaerobe* 59 38–48. 10.1016/j.anaerobe.2019.05.003 31102775

[B35] Neubauer V. Petri R. Humer E. Kröger I. Mann E. Reisinger N. (2018). High-grain diets supplemented with phytogenic compounds or autolyzed yeast modulate ruminal bacterial community and fermentation in dry cows. *J. Dairy Sci.* 101 2335–2349. 10.3168/jds.2017-13565 29331466

[B36] Nocek J. E. (1997). Bovine acidosis: implications on laminitis. *J. Dairy Sci.* 80 1005–1028. 10.3168/jds.s0022-0302(97)76026-0 9178142

[B37] Nocek J. E. Holt M. G. Oppy J. (2011). Effects of supplementation with yeast culture and enzymatically hydrolyzed yeast on performance of early lactation dairy cattle. *J. Dairy Sci.* 94 4046–4056. 10.3168/jds.2011-4277 21787940

[B38] Oba M. Mewis J. L. Zhining Z. (2015). Effects of ruminal doses of sucrose, lactose, and corn starch on ruminal fermentation and expression of genes in ruminal epithelial cells. *J. Dairy Sci.* 98 586–594. 10.3168/jds.2014-8697 25468705

[B39] Oksanen J. Guillaume Blanchet F. Friendly M. Kindt R. Legendre P. McGlinn D. (2018). *Vegan**: Community Ecology Package. R Package Version 2.5-1.* Available at: https://CRAN.R-project.org/package=vegan 10.3168/jds.2014-8697 (accessed October, 2018). 25468705

[B40] Peterson J. Garges S. Giovanni M. McInnes P. Wang L. Schloss J. A. (2009). The NIH human microbiome project. *Genome Res.* 19 2317–2323. 10.1101/gr.096651.109 19819907 PMC2792171

[B41] Petri R. M. Kleefisch M. T. Metzler-Zebeli B. U. Zebeli Q. Klevenhusen F. (2018). Changes in the rumen epithelial microbiota of cattle and host gene expression in response to alterations in dietary carbohydrate composition. *Appl. Environ. Microbiol.* 84:e0384-18. 10.1128/aem.00384-18 29654184 PMC5981066

[B42] Petri R. M. Pourazad P. Khiaosa-ard R. Klevenhusen F. Metzler-Zebeli B. U. Zebeli Q. (2017). Temporal dynamics of in-situ fiber-adherent bacterial community under ruminal acidotic conditions determined by 16S rRNA gene profiling. *PLoS One.* 12:e0182271. 10.1371/journal.pone.0182271 28763489 PMC5538656

[B43] Petri R. M. Schwaiger T. Penner G. B. Beauchemin K. A. Forster R. J. McKinnon J. J. (2013). Characterization of the core rumen microbiome in cattle during transition from forage to concentrate as well as during and after an acidotic challenge. *PLoS One* 8:e83424. 10.1371/journal.pone.0083424 24391765 PMC3877040

[B44] Petri R. M. Wetzels S. U. Qumar M. Khiaosa-ard R. Zebeli Q. (2019). Adaptive responses in short-chain fatty acid absorption, gene expression, and bacterial community of the bovine rumen epithelium recovered from a continuous or transient high-grain feeding. *J. Dairy Sci.* 102 5361–5378. 10.3168/jds.2018-15691 31005320

[B45] Pfaffl M. W. Tichopad A. Prgomet C. Neuvians T. P. (2004). Determination of stable housekeeping genes, differentially regulated target genes and sample integrity: BestKeeper – Excel-based tool using pair-wise correlations. *Biotechnol. Lett.* 26 509–515. 10.1023/b:bile.0000019559.84305.47 15127793

[B46] Plaizier J. C. Khafipour E. Li D. Gozho G. N. Krause D. O. (2012). Subacute ruminal acidosis (SARA), endotoxins and health consequences. *Anim. Feed Sci. Technol.* 172 9–21. 10.1016/j.anifeedsci.2011.12.004

[B47] Quast C. Pruesse E. Yilmaz P. Gerken J. Schweer T. Yarza P. (2012). The SILVA ribosomal RNA gene database project: improved data processing and web-based tools. *Nucleic Acids Res.* 41 D590–D596. 10.1093/nar/gks1219 23193283 PMC3531112

[B48] Qumar M. Khiaosa-ard R. Klevenhusen F. Plaizier J. C. Zebeli Q. (2017). Gastrointestinal endotoxin and metabolic responses in cows fed and recovered from two different grain-rich challenges. *Livest. Sci.* 1 120–123. 10.1016/j.livsci.2017.07.015

[B49] Schmittgen T. D. Livak K. J. (2008). Analyzing real-time PCR data by the comparative C T method. *Nat. Protoc.* 3 1101–1108. 10.1038/nprot.2008.73 18546601

[B50] Segata N. Izard J. Waldron L. Gevers D. Miropolsky L. Garrett W. S. (2011). Metagenomic biomarker discovery and explanation. *Genom. Biol.* 12:R60. 10.1186/gb-2011-12-6-r60 21702898 PMC3218848

[B51] Steele M. A. Croom J. Kahler M. AlZahal O. Hook S. E. Plaizier K. (2011). Bovine rumen epithelium undergoes rapid structural adaptations during grain-induced subacute ruminal acidosis. *Am. J. Physiol. Regul. Integr. Compar. Physiol.* 300 R1515–R1523. 10.1152/ajpregu.00120.2010 21451145

[B52] Steele M. A. Dionissopoulos L. AlZahal O. Doelman J. McBride B. W. (2012). Rumen epithelial adaptation to ruminal acidosis in lactating cattle involves the coordinated expression of insulin-like growth factor-binding proteins and a cholesterolgenic enzyme. *J. Dairy Sci.* 95 318–327. 10.3168/jds.2011-4465 22192211

[B53] van Gylswyk N. O. (1995). *Succiniclasticum ruminis* gen. nov., sp. nov., a ruminal bacterium converting succinate to propionate as the sole energy-yielding mechanism. *Int. J. Syst. Bacteriol.* 45 297–300. 10.1099/00207713-45-2-297 7537062

[B54] Wetzels S. U. Mann E. Pourazad P. Qumar M. Pinior B. Metzler-Zebeli B. U. (2017). Epimural bacterial community structure in the rumen of Holstein cows with different responses to a long-term subacute ruminal acidosis diet challenge. *J. Dairy Sci.* 100 1829–1844. 10.3168/jds.2016-11620 28041738

[B55] Zanello G. Berri M. Dupont J. Sizaret P.-Y. D’Inca R. Salmon H. (2011). *Saccharomyces cerevisiae* modulates immune gene expressions and inhibits ETEC-mediated ERK1/2 and p38 signaling pathways in intestinal epithelial cells. *PLoS One* 6:e18573. 10.1371/journal.pone.0018573 21483702 PMC3070739

[B56] Zebeli Q. Dijkstra J. Tafaj M. Steingass H. Ametaj B. Drochner W. (2008). Modeling the adequacy of dietary fiber in dairy cows based on the responses of ruminal pH and milk fat production to composition of the diet. *J. Dairy Sci.* 91 2046–2066. 10.3168/jds.2007-0572 18420634

[B57] Zebeli Q. Metzler-Zebeli B. U. (2012). Interplay between rumen digestive disorders and diet-induced inflammation in dairy cattle. *Res. Vet. Sci.* 93 1099–1108. 10.1016/j.rvsc.2012.02.004 22370295

