# Supplementary material for: Feed Additives Differentially Impact the Epimural Microbiota and Host Epithelial Gene Expression of the Bovine Rumen Fed Diets Rich in Concentrates
Source: Front Microbiol. 2020 Feb 19;11:119. doi: 10.3389/fmicb.2020.00119 (PMC7043141; doi:10.3389/fmicb.2020.00119)

**Supplemental Table 1.** Percent relative abundance of the 58 genera identified using 16S rRNA and the SILVA database.

|  | Treatment^1^ | | |  | |  |
| --- | --- | --- | --- | --- | --- | --- |
| Genera | CON | PHY | AY | SEM | *P*-value  Treatment | |
| *Achromobacter* | 0.030 | 0.058 | 0.043 | 0.0082 | | 0.56 |
| *Acinetobacter* | 0.002 | 0.000 | 0.002 | 0.0006 | | 0.52 |
| *Actinobaculum* | 0.015 | 0.026 | 0.013 | 0.0039 | | 0.60 |
| *Actinomyces* | 0.011 | 0.008 | 0.004 | 0.0021 | | 0.74 |
| *Agrobacterium* | 0.006 | 0.009 | 0.003 | 0.0016 | | 0.73 |
| *Akkermansia* | 0.013 | 0.010 | 0.000 | 0.0038 | | 0.47 |
| *Anaerostipes* | 0.000 | 0.003 | 0.000 | 0.0010 | | 0.40 |
| *Anaerovibrio* | 0.103 | 0.268 | 0.019 | 0.0731 | | 0.32 |
| *Anaerovorax* | 0.002 | 0.003 | 0.004 | 0.0005 | | 0.64 |
| *Arcanobacterium* | 0.002 | 0.003 | 0.003 | 0.0003 | | 0.89 |
| *Atopobium* | 0.077 | 0.103 | 0.065 | 0.0110 | | 0.46 |
| *Bacillus* | 0.008 | 0.017 | 0.016 | 0.0028 | | 0.67 |
| *Bifidobacterium* | 2.59 | 0.66 | 1.19 | 0.5761 | | 0.12 |
| *Blvii28* | 0.483 | 0.671 | 0.579 | 0.0543 | | 0.77 |
| *Butyrivibrio* | 4.18 | 4.98 | 5.30 | 0.3326 | | 0.45 |
| *Campylobacter* | 7.73 | 8.36 | 10.49 | 0.8334 | | 0.23 |
| *Clostridium* | 0.191 | 0.092 | 0.070 | 0.0374 | | 0.26 |
| *Collinsella* | 0.008 | 0.011 | 0.013 | 0.0014 | | 0.86 |
| *Coprococcus* | 0.004 | 0.009 | 0.016 | 0.0033 | | 0.55 |
| *Dehalobacterium* | 0.386 | 0.312 | 0.293 | 0.0283 | | 0.50 |
| *Delftia* | 0.012 | 0.027 | 0.015 | 0.0045 | | 0.62 |
| *Desulfobulbus* | 16.78 | 18.55 | 19.74 | 0.8607 | | 0.47 |
| *Desulfovibrio* | 3.21 | 2.89 | 2.58 | 0.1840 | | 0.56 |
| *Elusimicrobium* | 0.002 | 0.001 | 0.003 | 0.0004 | | 0.81 |
| *Lachnospira* | 0.001 | 0.002 | 0.001 | 0.0005 | | 0.33 |
| *Lactobacillus* | 0.136 | 0.139 | 0.083 | 0.0182 | | 0.70 |
| *Megasphaera* | 0.709 | 0.396 | 0.453 | 0.0964 | | 0.53 |
| *Methanimicrococcus* | 0.007 | 0.003 | 0.005 | 0.0011 | | 0.76 |
| *Methanobrevibacter* | 0.183 | 0.204 | 0.191 | 0.0059 | | 0.91 |
| *Methanosphaera* | 0.007 | 0.007 | 0.007 | 0.0001 | | 1.00 |
| *Moryella* | 0.004 | 0.010 | 0.006 | 0.0018 | | 0.58 |
| *Oscillospira* | 0.170 | 0.259 | 0.165 | 0.0304 | | 0.34 |
| *Phenylobacterium* | 0.052 | 0.050 | 0.017 | 0.0115 | | 0.14 |
| *Phyllobacterium* | 0.010 | 0.013 | 0.002 | 0.0035 | | 0.19 |
| *Pseudobutyrivibrio* | 0.007 | 0.016 | 0.004 | 0.0036 | | 0.06 |
| *Pseudoramibacter_Eubacterium* | 0.000 | 0.001 | 0.002 | 0.0005 | | 0.22 |
| *Pyramidobacter* | 0.061 | 0.038 | 0.043 | 0.0072 | | 0.37 |
| *Ralstonia* | 0.047 | 0.089 | 0.090 | 0.0141 | | 0.52 |
| rc4-4 | 0.004 | 0.001 | 0.023 | 0.0068 | | 0.43 |
| RFN20 | 0.010 | 0.010 | 0.021 | 0.0036 | | 0.61 |
| *Ruminobacter* | 0.071 | 0.074 | 0.071 | 0.0010 | | 1.00 |
| *Ruminococcus* | 1.00 | 0.87 | 1.09 | 0.0654 | | 0.84 |
| *Selenomonas* | 0.077 | 0.193 | 0.014 | 0.0524 | | 0.07 |
| *Sharpea* | 0.105 | 0.194 | 0.141 | 0.0258 | | 0.63 |
| SHD-231 | 0.020^a^ | 0.054^b^ | 0.024^a^ | 0.0107 | | 0.01 |
| *Shuttleworthia* | 0.040 | 0.054 | 0.035 | 0.0058 | | 0.73 |
| SJA-88 | 0.007 | 0.008 | 0.001 | 0.0021 | | 0.57 |
| *Slackia* | 0.016 | 0.001 | 0.001 | 0.0049 | | 0.19 |
| *Sphingomonas* | 0.039 | 0.066 | 0.055 | 0.0078 | | 0.56 |
| *Streptococcus* | 0.006 | 0.005 | 0.002 | 0.0011 | | 0.57 |
| *Succiniclasticum* | 9.18 | 6.10 | 5.49 | 1.1419 | | 0.07 |
| *Succinivibrio* | 0.298 | 0.071 | 0.157 | 0.0660 | | 0.19 |
| *Suttonella* | 0.970 | 1.043 | 0.810 | 0.0688 | | 0.84 |
| *Syntrophomonas* | 0.028 | 0.028 | 0.055 | 0.0090 | | 0.35 |
| TG5 | 0.569 | 0.785 | 0.613 | 0.0659 | | 0.24 |
| *Turicibacter* | 0.007 | 0.002 | 0.023 | 0.0063 | | 0.49 |
| VadinCA11 | 0.010 | 0.008 | 0.007 | 0.0011 | | 0.72 |
| *Veillonella* | 0.249 | 0.010 | 0.000 | 0.0813 | | 0.16 |

SEM: standard error of the mean

^1^Treatment is the additive that was added to SARA diets; CON: control, no supplementation; PHY: phytogenic product; AY: autolyzed yeast.

^a,b^ Differing superscripts in the same row indicate significant variation in comparison to the control group based on Dunnett-Hsu analysis.

**Supplemental Table 2.** Significantly impacted OTUs based on feed additive supplementation with identification of nearest relative to 97% identity based on the RDP SILVA v123 database. Taxa are sorted based on relative abundance.

|  | | | Total Relative Abundance | Treatment^1^ | | | |  | | *P* - value | |
| --- | --- | --- | --- | --- | --- | --- | --- | --- | --- | --- | --- |
| Phylum | Family | Genera |  | CON | PHY | AY | SEM | | Treatment | |  |
| *Firmicutes* | *Ruminococcaceae* |  | 13.20% | 10.65 | 11.60 | 11.89 | 0.004 | | 0.78 | |  |
| *Proteobacteria* | *Comamonadaceae* |  | 7.95% | 5.66 | 7.24 | 5.93 | 0.005 | | 0.46 | |  |
| *Proteobacteria* | *Neisseriaceae* |  | 3.15% | 2.45 | 2.35 | 2.37 | 0.0003 | | 0.98 | |  |
| *Firmicutes* | *Veillonellaceae* | *Succiniclasticum* | 2.54% | 4.03^b^ | 2.60^a^ | 1.95^a^ | 0.006 | | 0.05 | |  |
| *Actinobacteria* | *Coriobacteriaceae* |  | 1.81% | 2.51 | 1.96 | 1.72 | 0.002 | | 0.75 | |  |
| *Firmicutes* | *Veillonellaceae* | *Succiniclasticum* | 1.78% | 1.90 | 1.20 | 0.91 | 0.003 | | 0.28 | |  |
| *Proteobacteria* | *Campylobacteraceae* | *Campylobacter* | 1.61% | 1.51 | 1.81 | 2.22 | 0.002 | | 0.08 | |  |
| *Firmicutes* | *Ruminococcaceae* |  | 1.47% | 1.29 | 1.07 | 1.55 | 0.001 | | 0.27 | |  |
| *Firmicutes* | *Ruminococcaceae* |  | 1.35% | 1.53 | 1.25 | 1.94 | 0.002 | | 0.66 | |  |
| *Firmicutes* | *Ruminococcaceae* |  | 1.20% | 0.875 | 1.02 | 1.27 | 0.0011 | | 0.20 | |  |
| *Firmicutes* | *Christensenellaceae* |  | 1.19% | 0.627 | 1.05 | 1.28 | 0.0019 | | 0.13 | |  |
| *Actinobacteria* | *Bifidobacteriaceae* | *Bifidobacterium* | 1.17% | 1.60 | 0.373 | 0.985 | 0.0035 | | 0.37 | |  |
| *Proteobacteria* | *Cardiobacteriaceae* | *Suttonella* | 1.13% | 1.42 | 1.99 | 1.45 | 0.002 | | 0.66 | |  |
| *Firmicutes* |  |  | 1.08% | 1.37 | 0.877 | 1.27 | 0.0015 | | 0.35 | |  |
| *Firmicutes* |  |  | 1.08% | 1.10 | 1.12 | 0.788 | 0.0011 | | 0.72 | |  |
| *Firmicutes* |  |  | 1.01% | 1.77^b^ | 0.814^a^ | 0.652^a^ | 0.0035 | | 0.01 | |  |
| *Actinobacteria* | *Actinomycetaceae* |  | 0.95% | 1.33 | 0.574 | 0.721 | 0.0023 | | 0.57 | |  |
| *Firmicutes* | *Ruminococcaceae* |  | 0.80% | 0.859 | 0.911 | 0.630 | 0.0009 | | 0.48 | |  |
| *Firmicutes* | *Ruminococcaceae* | *Ruminococcus* | 0.67% | 0.990 | 1.14 | 1.217 | 0.0007 | | 0.92 | |  |
| *Actinobacteria* | *Bifidobacteriaceae* |  | 0.62% | 0.740 | 0.790 | 0.356 | 0.0014 | | 0.69 | |  |
| *Firmicutes* | *Ruminococcaceae* |  | 0.61% | 0.205 | 0.530 | 0.369 | 0.0009 | | 0.51 | |  |
| *Firmicutes* | *Ruminococcaceae* |  | 0.61% | 0.365 | 0.529 | 0.438 | 0.0005 | | 0.53 | |  |
| *Firmicutes* |  |  | 0.52% | 0.488 | 0.574 | 0.422 | 0.0004 | | 0.60 | |  |
| *Firmicutes* | *Ruminococcaceae* |  | 0.51% | 0.466 | 0.516 | 0.426 | 0.0003 | | 0.46 | |  |
| *Firmicutes* | *Christensenellaceae* |  | 0.48% | 0.428 | 0.414 | 0.509 | 0.0003 | | 0.67 | |  |
| *Firmicutes* | *Lachnospiraceae* | *Butyrivibrio* | 0.41% | 0.440 | 0.673 | 0.397 | 0.0009 | | 0.15 | |  |
| *Firmicutes* | *Veillonellaceae* | *Succiniclasticum* | 0.38% | 0.438 | 0.459 | 0.344 | 0.0004 | | 0.85 | |  |
| *Firmicutes* | *Lachnospiraceae* |  | 0.37% | 0.579 | 0.403 | 0.374 | 0.0006 | | 0.31 | |  |
| *Firmicutes* | *Ruminococcaceae* |  | 0.37% | 0.345 | 0.317 | 0.333 | 0.0001 | | 0.95 | |  |
| *Firmicutes* | *Ruminococcaceae* |  | 0.33% | 0.255 | 0.196 | 0.373 | 0.0005 | | 0.06 | |  |
| *Proteobacteria* | *Comamonadaceae* |  | 0.32% | 0.290 | 0.310 | 0.246 | 0.0002 | | 0.51 | |  |
| *Firmicutes* | *Ruminococcaceae* |  | 0.32% | 0.264 | 0.259 | 0.305 | 0.0001 | | 0.80 | |  |
| *Synergistetes* | *Dethiosulfovibrionaceae* | TG5 | 0.30% | 0.314 | 0.353 | 0.274 | 0.0002 | | 0.67 | |  |
| *Firmicutes* | *Ruminococcaceae* |  | 0.30% | 0.200^a^ | 0.454^b^ | 0.265^a^ | 0.0008 | | 0.04 | |  |
| *Firmicutes* |  |  | 0.29% | 0.269 | 0.372 | 0.315 | 0.0003 | | 0.72 | |  |
| *Firmicutes* | *Veillonellaceae* | *Succiniclasticum* | 0.29% | 0.440 | 0.341 | 0.288 | 0.0004 | | 0.58 | |  |
| *Firmicutes* | *Ruminococcaceae* |  | 0.26% | 0.182 | 0.165 | 0.294 | 0.0004 | | 0.18 | |  |
| *Firmicutes* | *Ruminococcaceae* |  | 0.26% | 0.302 | 0.097 | 0.388 | 0.0009 | | 0.24 | |  |
| *Firmicutes* | *Veillonellaceae* | *Megasphaera* | 0.24% | 0.369 | 0.169 | 0.321 | 0.0006 | | 0.50 | |  |
| *Synergistetes* | *Dethiosulfovibrionaceae* | TG5 | 0.24% | 0.142^a^ | 0.366^b^ | 0.175^a^ | 0.0007 | | 0.01 | |  |
| *Firmicutes* |  |  | 0.23% | 0.464 | 0.250 | 0.278 | 0.0007 | | 0.17 | |  |
| *Firmicutes* | *Veillonellaceae* |  | 0.20% | 0.360 | 0.138 | 0.063 | 0.0009 | | 0.10 | |  |
| *Proteobacteria* | *Pelobacteraceae* |  | 0.19% | 0.154 | 0.186 | 0.187 | 0.0001 | | 0.87 | |  |
| *Synergistetes* | *Dethiosulfovibrionaceae* | TG5 | 0.19% | 0.119 | 0.235 | 0.169 | 0.0003 | | 0.18 | |  |
| GN02 |  |  | 0.19% | 0.123 | 0.174 | 0.158 | 0.0001 | | 0.48 | |  |
| *Synergistetes* | *Dethiosulfovibrionaceae* | TG5 | 0.18% | 0.118 | 0.172 | 0.124 | 0.0002 | | 0.40 | |  |

SEM: standard error of the mean

^1^Treatment is the additive that was added to SARA diets; CON: control, no supplementation; PHY: phytogenic product; AY: autolyzed yeast.

^a,b^ Differing superscripts in the same row indicate significant variation in comparison to the control group based on Dunnett-Hsu analysis.

**Supplemental Figure 1.** Rarefaction curves indicating the good coverage values for depth of analysis up to 344 sequences per sample for both additive (A) and day (B).


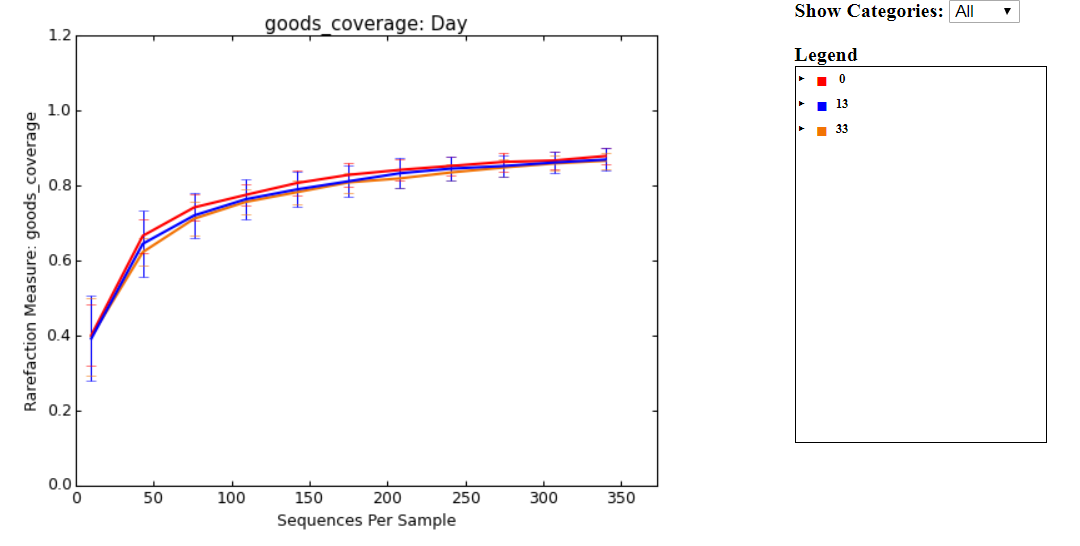

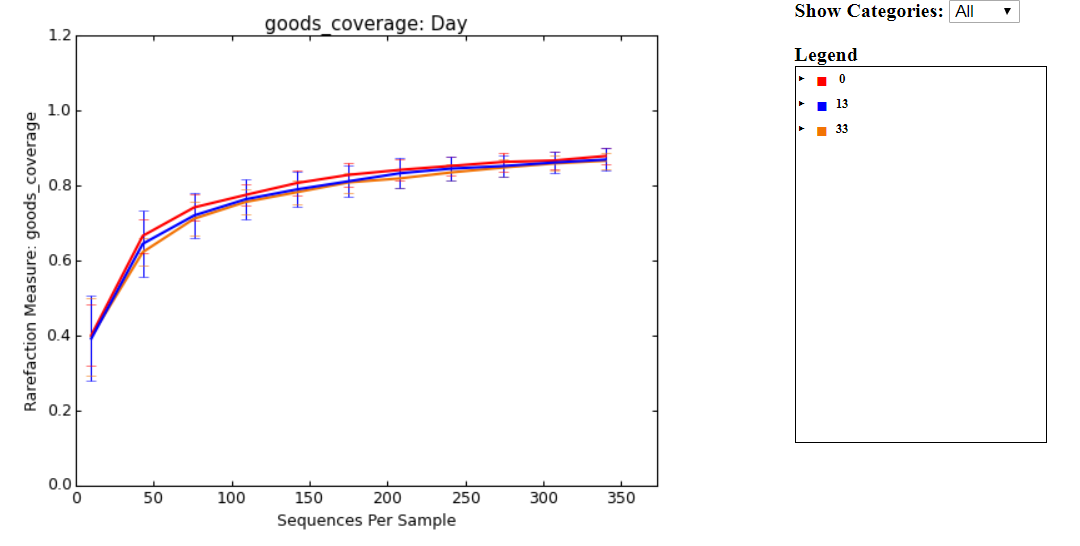

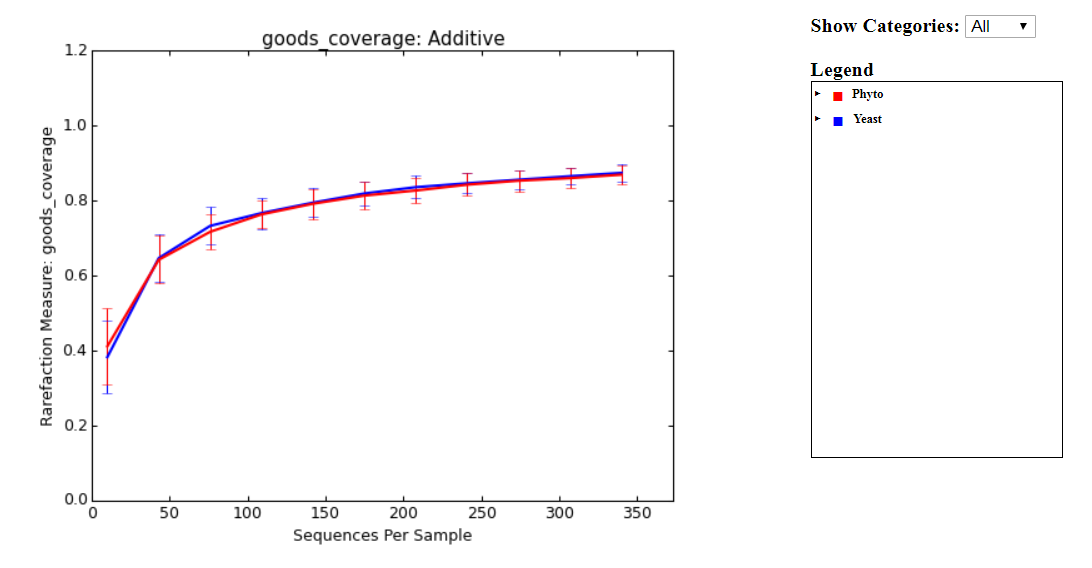

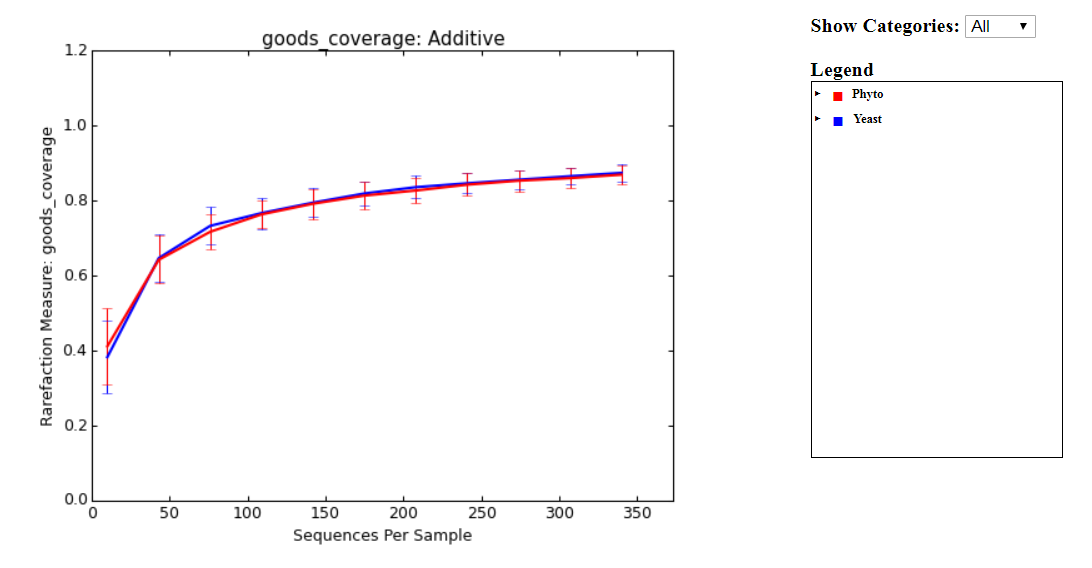


B

A

**Supplemental Figure 2.** Whisker-plots describing the impact of feed additives on the various alpha diversity indices (number of observations, Simpsons index, Chao1 index, Shannon index) for the rumen microbial population under high grain feeding. Feed additives are: Control: white boxes; Phytogenic: light grey boxes; Autolyzed Yeast: dark grey boxes. The thick black line in each box represents the median for each indices. Whiskers represent the upper and lower quartiles of deviation from the median. The inter-quartile range represents the middle 50% of the index values.


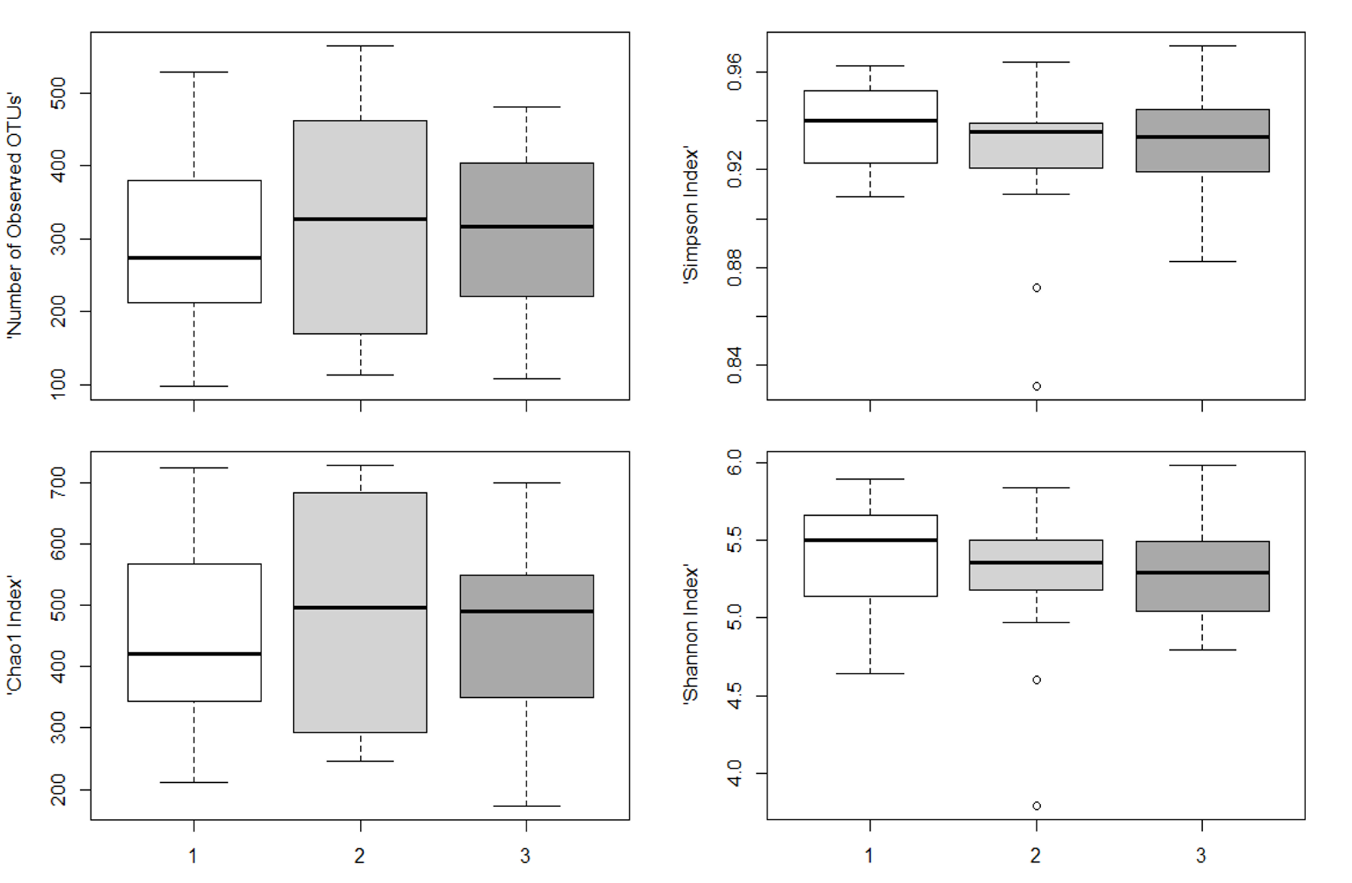

Supplement: Supplementary file 1 [file Data_Sheet_1.docx]
